# Local Interactions of Atmospheric Oxygen with MoS_2_ Crystals

**DOI:** 10.3390/ma14205979

**Published:** 2021-10-11

**Authors:** Robert Szoszkiewicz

**Affiliations:** Faculty of Chemistry, Biological and Chemical Research Centre, University of Warsaw, Żwirki i Wigury 101, 02-089 Warsaw, Poland; rszoszkiewicz@chem.uw.edu.pl

**Keywords:** MoS_2_ crystals, α-MoO_3_, surface oxidation, surface etching

## Abstract

Thin and single MoS_2_ flakes are envisioned to contribute to the flexible nanoelectronics, particularly in sensing, optoelectronics and energy harvesting. Thus, it is important to study their stability and local surface reactivity. Their most straightforward surface reactions in this context pertain to thermally induced interactions with atmospheric oxygen. This review focuses on local and thermally induced interactions of MoS_2_ crystals and single MoS_2_ flakes. First, experimentally observed data for oxygen-mediated thermally induced morphological and chemical changes of the MoS_2_ crystals and single MoS_2_ flakes are presented. Second, state-of-the-art mechanistic insight from computer simulations and arising open questions are discussed. Finally, the properties and fate of the Mo oxides arising from thermal oxidation are reviewed, and future directions into the research of the local MoS_2_/MoO_x_ interface are provided.

## 1. Introduction

**Figure 1:**
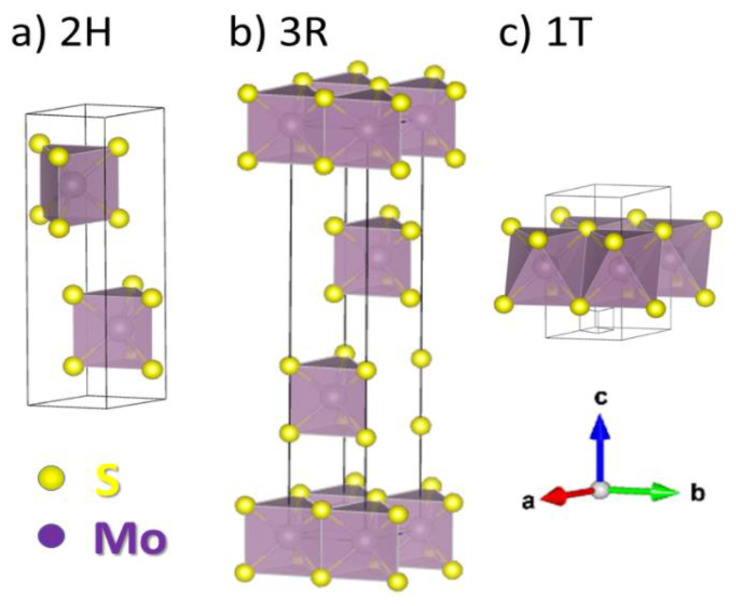
Crystalline structures of the MoS_2_ polymorphs. Each MoS_2_ layer is a stack comprising an S plane on a Mo plane on yet another S plane. (**a**) The most common 2H polymorphic structure with a hexagonal symmetry, two layers per repeat unit and Mo trigonal prismatic coordination; (**b**) 3R structure with rhombohedral symmetry, three layers per repeat unit and Mo trigonal prismatic coordination; (**c**) 1T structure with tetragonal symmetry, one layer per repeat unit and Mo octahedral coordination (same as in the most stable oxides and molybdates). The lattice constant “a” is 0.31 nm [1], and the thickness of a single MoS_2_ layer is between 0.65 and 0.7 nm, depending on the particular substrate.

MoS_2_ belongs to a class of transition metal dichalcogenides (TMDCs). TMDCs share a common formula MeX_2_, where Me is a transition metal element from group four (Ti, Zr, Hf), five (V, Nb or Ta) or six (Mo, W), and X is a chalcogen (S, Se or Te). Their crystalline structure comprises an inner Me layer sandwiched by two X layers. Owing to two types of the Mo coordination and various arrangements of the S-Mo-S layers, there are three MoS_2_ polymorphs found in nature, with the 2H MoS_2_ structure being the most abundant (see Figure 1).

Initially, microscopic and naturally occurring MoS_2_ crystals, aka molybdenites, were used as solid lubricants in various encapsulated devices, such as low Earth orbit (LEO) satellites [2]. This is thanks to their low friction and propensity to peel off easily, because of any two adjacent layers interacting weakly via the van der Waals forces [3,4,5]. Later in time, microscopic MoS_2_ crystallites and their composites found usage in batteries, photovoltaic devices and catalysts for the hydrogen evolution reaction (HER) and other catalytic processes [6,7,8,9,10]. In recent years, interest in single MoS_2_ crystals has surged in the context of nanoelectronics, particularly on flexible substrates. According to the Web of Science, a number of published papers with the “MoS_2_” keyword increased slowly from 164 papers in 2000 to 220 papers in 2010, but then it started to grow exponentially from 288 papers in 2011 to reach 5549 papers in 2019 and level off to 5661 manuscripts published in 2020. Coincidently, year 2011 marked appearance of the first published reports about single-layer MoS_2_ transistors [11].

Most of the current research with MoS_2_ crystals has been performed on the 2H MoS_2_ polymorph. Moreover, 2H MoS_2_ has been shown to be a 2D semiconductor with high electron mobilities of up to 200 cm^2^ V^−1^ s^−1^ and a bulk 1.2 eV indirect bandgap, which increases and changes its character with decreasing number of the MoS_2_ layers, so that the 2H MoS_2_ monolayer has a 1.8 eV direct bandgap [3]. These properties make single 2H MoS_2_ flakes of variable thickness an ideal material for tightly packed nanoscale transistors and devices with variable bandgap. Recently, single-layer MoS_2_ based transistors, as well as their assemblies in the form of logic circuits [12,13,14] and in-memory computing devices with room-temperature current on/off ratios of 1 × 10^8^ and ultralow standby power dissipation in single layers of MoS_2_, have been developed [15,16]. At the same time, great progress has been made into synthesis of wafer-scale polycrystalline MoS_2_ monolayers [17] and large-domain (≈500 μm) single-crystalline MoS_2_ monolayers by chemical vapor deposition [14,18], as well as the wafer-scale transfer and stacking of monolayer MoS_2_ for heterogeneous integrations [19,20]. Due to discovery of large photoluminescence quantum efficiency of the chemically treated MoS_2_ crystals [21], next-generation flexible nanoelectronics devices based on single MoS_2_ flakes appear already in sensing, optoelectronics and energy harvesting [3,22,23]. Furthermore, the presence of various conduction mechanisms beyond electronic currents, such as spin currents, polariton currents, valley and other topological currents within single MoS_2_ layers and/or their heterostructures with other 2D materials, has opened new vistas in the MoS_2_ based nanoelectronics [3,24,25].

Each microelectronic device heats up while working, mostly due to Joule heating, and some MoS_2_ based transistors have been measured recently to locally reach 250 °C and more [26]. In addition, exposure to ambient conditions has been shown to reversibly reduce the on-state current in back-gated bilayer MoS_2_ based FETs by up to two orders of magnitude [27]. However, other studies on thin MoS_2_ FET transistors have shown up to three-fold drops in carrier mobilities in air with respect to vacuum, which did not recover upon vacuum annealing [28]. Therefore, it is important to investigate the chemical surface reactivity of single MoS_2_ flakes in ambient conditions above room temperature. Furthermore, the impact of relative humidity cannot be neglected due to capillary condensation at local lengths [29,30].

From the time of seminal works of Ross et al. [31] on Mo polycrystalline powders in the 1950s till about the 2010s MoS_2_ crystals have been considered inert to oxidation till 600 °C in air [32]. Substantial progress has been made in this field, particularly after 2013, when several seminal papers about local oxidative etching have been published [33,34,35]. A whole zoo of the surface reactions of 2H MoS_2_ crystals with oxygen, much beyond oxidative etching, has been discovered by using high-resolution tools, such as AFMs, SEMs and TEMs. These reactions involve physio and chemical oxygen adsorption, oxygen dissociative reactions, and other direct and non-direct oxidation mechanisms, as well as oxygen penetration between the stacked MoS_2_ layers [33,35,36,37,38,39,40,41,42,43]. However, despite substantial efforts interactions of oxygen with single MoS_2_ flakes and crystals are still not well understood, particularly in terms of several competing thermal etching mechanisms. Finally, it is also important to learn what kinds of Mo oxides are produced and in what morphologies they remain on the MoS_2_ surface, e.g., clusters/particles, dendrites, islands or even complete layers.

Consequently, this review summarizes the current state of the field and focuses on thermally induced interactions with oxygen of both thick and thin 2H MoS_2_ crystallites, as well as their thin flakes, down to the single 2H monolayers and nanosheets. The research performed in ambient and humid environments is reviewed together with the behavior of the MoS_2_ in selected liquids. The emphasis is on the structural data, if these are available in the original studies. Phenomenological observations and the mechanisms of these processes obtained mostly via computer simulations are detailed upon in Section 2 and Section 3, respectively. Various MoO_x_ oxides, their derivatives and properties as well as future directions of research in the MoS_2_/MoO_x_ interface are reviewed in Section 4. It is an interesting interface due to semiconducting character of the involved species, tunable MoS_2_ bandgap depending on its thickness and yet unclear semiconducting properties of the thin Mo oxide layers.

## 2. Phenomenological Observations of Thermal MoS_2_ Oxidation in Air and in Water

To start with, one must acknowledge major differences between MoS_2_ powders, bulk MoS_2_ crystals and single MoS_2_ flakes. Pulverized MoS_2_ powders comprise mostly the 2H MoS_2_ polymorph, and their physicochemical properties have been studied macroscopically. Bulk MoS_2_ crystals have been studied on the macro- and micro-scales, but mostly computationally. Single microscopic and exclusively 2H MoS_2_ crystalline flakes of various thickness from one monolayer (ML) to much thicker structures have been studied computationally, as well as experimentally. Research in these different MoS_2_ forms and such different length scales have provided certain amount of information at each length scale, which is often not directly transferable to a different length scale.

**Figure 2:**
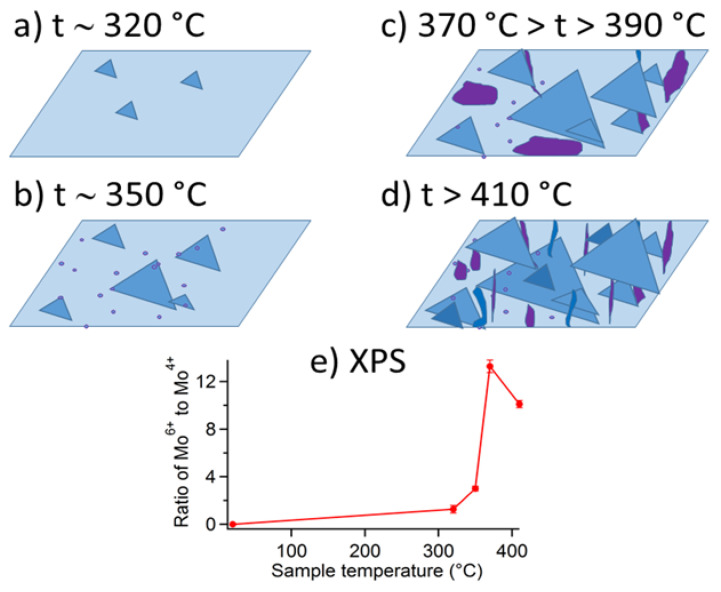
Phenomenological aspects of the MoO_x_ formation onto MoS_2_ basal planes during their heating in dry air. (**a**) Above ca. 320 °C, triangular 1 ML deep etch pits were observed [33,34,35,40]. (**b**) Nucleated growth of the etch pits was supplemented by appearance of small sub-nm size MoO_x_ clusters detected already at ca. 350 °C via a combination of various experimental techniques [47]. (**c**) At temperatures between 370 and 390 °C, the largest amounts of Mo oxides and their derivatives accumulate on the MoS_2_ surface [42]. Some of it forms irregular patches (marked in violet) [34,42]. (**d**) Above 410 °C, less oxide has been detected indirectly and locally via AFM techniques, as well as chemically and globally via XPS studies. In addition, in this regime, substantial surface defects have been observed. (**e**) Unpublished analysis based on the XPS results (from Reference [42]) collected on Si substrates with sparsely distributed and mechanically exfoliated 2H MoS_2_ flakes. It suggests the largest amount of the surface presents MoO_3_ oxides in the vicinity of 370 °C.

Early experimental studies of polycrystalline MoS_2_ powders have shown that Mo oxide layers present on the surface form slowly and act as passivating layers till at least 100 °C [31,35]. However, freshly exfoliated single MoS_2_ flakes did not show any direct manifestations of the protective oxide layers [44]. They displayed electronic density shifts within the MoS_2_ above 200 °C [35]. At temperatures between 320 and 400 °C oxidative thermal etching regime has been observed in the case of single MoS_2_ flakes [33,34,35,40]. Above 400–410 °C substantial oxidation involving decrease of the flakes’ volume has been observed in air [41,45]. It confirmed predictions about particularly fast oxidation along the crystalline edges [35,41,42,46]. Oxidation and oxidative etching in some instances produced visible MoO_3_ deposits (in various forms) appearing on the single MoS_2_ flakes/nanosheets [42,47,48] or even full MoS_2_ transformation [35] into single MoO_3_ crystals. The phenomenological aspects related to the MoO_x_ formation on globally heated basal planes within the MoS_2_ flakes in dry air are summarized in Figure 2.

Finally, additions of appreciable amounts of water vapors and/or water liquids have been shown to change everything and produce larger amounts of Mo oxides and their various forms then oxidation in air [31,36,42]. Below we discuss the aforementioned phenomena in more detail, as well as other related phenomena that are seen at various temperatures.

### 2.1. Interactions of the MoS_2_ Samples with Oxygen till 300 °C

**MoS_2_ polycrystalline powders are slowly passivated by Mo oxides at temperatures up to at least 100 °C.** Already in 1953–1955, Ross et al. published several reports on the existence of the MoO_3_ at low heating temperatures on powdered and pulverized macroscopic MoS_2_ samples originating from crystalline molybdenites [31,49]. Initially, Ross et al. used only potentiometric detection of surface acidity resulting both from Mo oxides and sulfates, since both sulfur and the molybdenum atoms can be oxidized. Later, to reduce interference from sulfates, they produced colorimetric detection tests of molybdenum thiocyanate complex derived from MoO_3_. The acidity measurements suggested the existence of MoO_3_ being only about one monolayer thick. Furthermore, no measurable increase of surface acidity in MoS_2_ samples kept at room temperature for 20 months was observed. This suggested stable MoS_2_ passivation at room temperature, achieved with a very thin MoO_3_ passivation layer.

For the powdered MoS_2_ samples, which were chemically stripped from a protective oxide layer due to a prolonged incubation in concentrated ammonia, Ross et al. measured their macroscopic oxidation rates at 100 °C in dry air and at 85 °C in humid air [31,49]. Humid air was produced with water vessel placed within the oven, but its actual relative humidity at 85 °C was not measured. A maximum percentage of such produced oxide was at most corresponding to about 1/3 of the MoO_3_ monolayer for oxidation times of up to ca. 200 h. They also observed a 10-fold increase of the MoS_2_ surface acidity for samples heated at 110 °C in air for 45 days, which corresponded to creation of up to several MoO_3_ monolayers. They reported as well that humid air oxidized the samples several times faster than air. In either case, the MoO_3_ layer formed very slowly, particularly at room temperature.

From the experiments on the single MoS_2_ flakes (see respective paragraphs below) the MoO_3_ presence already at room and low heating temperatures can be understood only as appearing mostly due to abounding presence of easily oxidized edges and defects within the MoS_2_ powdered crystallites. However, stable MoO_3_ passivation is understandable due to stable and dense structure of the most stable α-MoO_3_ (molybdite) comprising two well-interconnected layers of the MoO_6_ octahedra (see Figure 19, in Section 4) [50,51]. Such exceptional stability of the protective MoO_3_ layer at ambient conditions has been confirmed by other non-directly related studies. First, it has been observed that transparent MoO_3_ crystals change into yellow and grayish blue, only when oxygen defects are forcefully introduced, e.g., via stimulated hydrogen adsorption [50]. Second, XPS and XRD experiments on thin Mo films have shown that all defective MoO_3_ species with Mo^5+^ and Mo^4+^ oxidized to MoO_3_ already at 5% of oxygen content in reactive gases [52].

**Figure 3:**
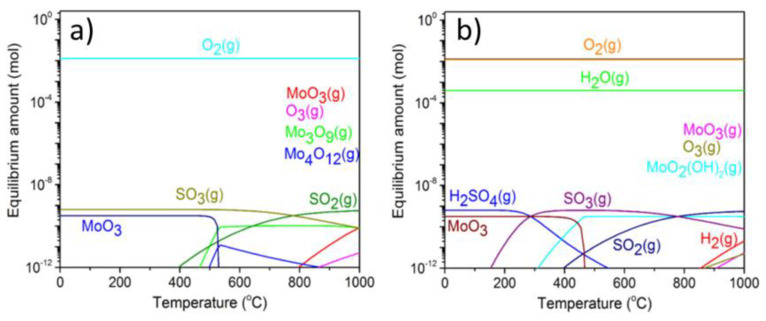
Thermodynamic calculations by Walter et al. showing thermal oxidation products for a fixed volume of the MoS_2_ crystal placed within a closed container at partial pressure of oxygen, similar to air, (**a**) and with water vapors (**b**). Reprinted from Reference [36], with permission. Copyright 2017, American Vacuum Society.

**Thermodynamic calculations show that bulk MoS_2_ crystals converts exclusively into MoO_3_ in dry air, but other Mo oxide species appear in humid air.** By performing thermodynamic calculations and using enthalpies of bulk reactions, Walter et al. obtained that within a closed system all of the MoS_2_ is expected to transform into the MoO_3_ species already at room temperature. Same outcome was obtained at any temperature above room temperature until a sublimation temperature of bulk MoO_3_, which was about 470 °C (see Figure 3a) [36]. Above 470 °C, volatile (MoO_3_)_x_ (x > 3) species started to appear. At 525 °C none of the MoO_3_ was predicted to stay on the oxidized MoS_2_ samples.

Somewhat different MoS_2_ oxidation outcome has been obtained by Walter et al. in the presence of humid air (see Figure 3b). For calculations therein, partial pressure of water vapors was set to the saturated water vapor pressure at room temperature, or to 100% relative humidity at 25 °C (298 K). Due to a saturated water pressure being a steeply increasing function above 200 °C, this corresponds to almost zero relative humidity above 300 °C. Nevertheless, what apparently mattered were initially added water molecules, which yielded additional Mo oxidized species. Not only MoO_3_, but also volatile molybdenum (VI) hydroxy-oxides, MoO_2_(OH)_2_(g), were predicted to appear above 300 °C in the calculations of Walter et al. in water vapors. It is not clear whether MoO_2_(OH)_2_ originated directly from water-mediated oxidation of the MoS_2_ crystals or rather from water reacting with the MoO_3_ adsorbed already on the MoS_2_ crystals, which is also likely, although has not been proven [42].

Smolik et al. reported experimental data and thermodynamical calculations on oxidation, volatilization and re-deposition of molybdenum oxide species formed from a certain molybdenum alloy heated between 400 and 800 °C in flowing dry and humid air [53]. Their conclusions agreed with the simulations of Walter et al., as well as their own simulations based on similar data as the ones of Walter et al. The Mo oxide species on Mo alloy underwent volatilization, which was dominated by the appearance of MoO_3_ above 550 °C and by the appearance of the MoO_2_(OH)_2_, formed from the small ingress of water vapor, at temperatures below 550 °C. All of these products were measured in the exhaust gases.

In conclusion, bulk thermodynamic simulations predict a full conversion of the MoS_2_ crystals into MoO_3_(g) as well as appearance of some polyoxides in dry air. However, existence of other volatile Mo species, such as MoO_2_(OH)_2_, has been predicted and observed in humid air. These results provide solid basis for discussion of the MoS_2_ oxidation, particularly at elevated temperatures.

**Single non-defective MoS_2_ flakes do not oxidize spontaneously.** There have been at least several studies tackling aging of single MoS_2_ flakes for a prolonged time in air. For example, Mirabelli et al. did not observe any aging over 27 days for mechanically exfoliated geological 2H MoS_2_ in air [54]. Their AFM topography results yielded pretty constant RMS roughness values of 0.2–0.4 nm throughout the 27 days after exfoliation. However, no other techniques, such as XPS or photoluminescence, have been used therein to confirm the lack of any passivating oxide layer on their samples. Similar conclusions have been reached by Jo et al. in their experimental and simulation study of mechanically cleaved geological MoS_2_ microflakes [55]. Experimentally, Jo et al. wanted to quantify the oxide growth rate using spectroscopic ellipsometry (SE) and found that their pristine MoS_2_ surface did not spontaneously oxidize in air within their experimental time scale of 6 days. Their SE modeling and data analysis predicted a constant oxide thickness of ca. 1 Å, which is much less than thickness of a single MoO_3_ layer. Such an outcome was obtained 5 h after cleaving, and it remained constant for the time of measurements, which was about 6 days. In addition, they did XPS studies on MoS_2_ crystals stored at ambient conditions after a year from its initial mechanical cleavage and did not detect any MoO_3_ species either, i.e., the Mo^6+^ states have been missing in their Mo 3d XPS spectra.

In contrast, Gao et al. [56] showed that chemical vapor deposition (CVD)-grown MoS_2_ monolayers were very air-sensitive. Their XPS and Auger electron spectroscopy studies conducted for about a year revealed gradual oxidation with time, which initiated from the crystal edges and propagated into the basal planes. In particular, their calculated percentage of the Mo^6+^ states within the XPS spectra of the Mo 3d electronic states increased from 14% for the 6-month-old sample to 35% for the 1-year-old sample. Next, they used photoluminescence studies to carry out “accelerated aging tests”, i.e., 20 min heating in 80 °C and at relative humidity of 65%. They compared mechanically exfoliated MoS_2_ monolayers and CVD-grown MoS_2_ monolayers, both on SiO_2_/Si substrates. The results indicated that mechanically exfoliated MoS_2_ samples showed only slight signs of ageing when compared to the CVD-grown MoS_2_ samples. This suggested that a large presence of S vacancies along the grain boundaries in the CVD-grown samples played a crucial role in oxidation.

**Room temperature interactions of basal MoS_2_ planes with oxygen produce defects, which can be oxidized, but not towards MoO_3_.** While computational approaches to MoS_2_ oxidation are presented later on, their main conclusions are that O_2_ molecules in contrast to molecular oxygen do not want to react with S atoms on a pristine, non-defective MoS_2_ surface [43,57,58,59]. Nevertheless, a defect-free character of single MoS_2_ crystals in ambient conditions results has been questioned by Peto et al. [60], who showed single oxidation events and high molecular oxygen affinity to the omnipresent sulfur vacancies on the MoS_2_ basal planes. Their freshly prepared 2H geological MoS_2_ crystals contained a native point defect density in the range of 1 × 10^11^ to 1 × 10^12^ cm^−2^. After a month of ambient exposure their STM measurements revealed formation of new point defects in basal planes, increasing the defect concentration 10-fold into the 3 × 10^12^ to 2 × 10^13^ cm^−2^ range. After a year of ambient exposure, they obtained yet another 10-fold increase in the defect concentration, now to 5 × 10^13^ to 1 × 10^14^ cm^−2^. The studies of Peto et al. did not find any direct evidence for the MoO_3_ present on such samples. They found that defects reacted readily with atomic and molecular oxygen to form substitutional oxygen species, i.e., oxygen atoms directly connected to Mo atoms.

**Computational studies suggest that MoO_3_ can appear on crystal edges already at room temperature.** The oxidation at room temperature has been suggested to proceed easily on MoS_2_ edges, as well as on grain boundaries and under-coordinated atomic sites via either oxygen substitutional doping (therein: barrier-less direct bonding with Mo atoms via Mo dangling bonds) [57] or via low-barrier dissociative oxygen adsorption (therein: O-S bonds formation with DFT calculated activation energy of only 0.3 eV) [58]. Martincová et al. [58] suggested that dissociative oxygen molecule splitting (viewed as catalyzed by an MoS_2_ edge) leads to substitutional binding of oxygen atoms to Mo atoms via a mechanism involving several steps and in consequence formations of the structures resembling MoO_3_ structures. Details of their proposed mechanism are discussed in Section 3 of this review. Chemical inertness of the basal MoS_2_ flakes towards interactions with oxygen and postulated relative easiness for such reactions at the crystal edges resemble the conclusions obtained in the case of HER catalyzed by MoS_2_. HER studies revealed that only crystalline edges were active, while the basal planes were inert [8].

**Figure 4:**
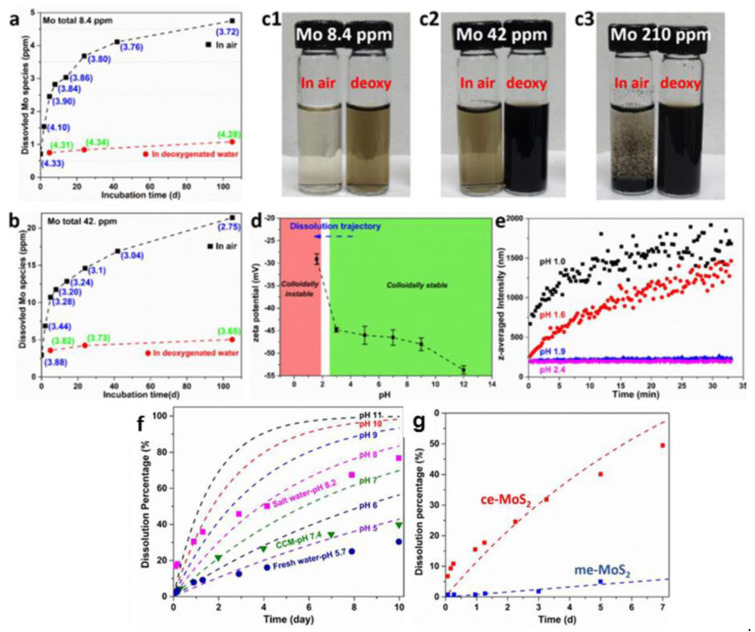
Very defective liquid-exfoliated nanosheets from the MoS_2_ powder (ce-MoS_2_) formed colloidal solution and dissolved in water, when at pH > 2 and at Mo concentrations below 2.2 mM. Less defective, ultrasonically exfoliated nanosheets from the MoS_2_ powder (ue-MoS_2_) dissolved much less. (**a**) Dissolution of ce-MoS_2_ nanosheets at low [Mo] of 8.4 ppm corresponding to 0.09 mM Mo in both air-saturated and deoxygenated water. Measured solution pH values are provided in parentheses for each data point. Upon ce-MoS_2_ dissolution pH decreases. (**b**) Same, at [Mo] of 42 ppm (0.44 mM Mo). Larger pH decrease was observed upon ce-MoS_2_ dissolution than in (**a**). (**c**) Photographs showing that ce-MoS_2_ ions were colloidally stable in all dispersions after 105-day incubation, except at the highest starting Mo concentrations (210 ppm or 2.2 mM) in O_2_-containing water. Aggregation of the ce-MoS_2_ below pH of 1.9 was confirmed by measurements of zeta potentials (**d**) and hydrodynamic sizes (**e**) at various pH values. Dynamic light scattering was used to measure the change, and the pH of the solution was adjusted by addition of HCl or NaOH to ce-MoS_2_ solution (∼10 ppm of Mo). (**f**) pH-dependent dissolution rates obtained by using various buffer solutions. The lines showed results of an empirical kinetic law. (**g**) Dissolution rates for chemically vs. ultrasonically exfoliated MoS_2_ nanosheets in HEPES buffers (pH 7) at [Mo] of 8.4 ppm. Both kinds of MoS_2_ nanosheets underwent continuous dissolution, but the kinetics was much slower for the ultrasonically exfoliated samples. Reprinted from Reference [61], with permission. Copyright 2016 American Chemical Society.

**Thin and defected MoS_2_ nanosheets dissolve in water at room temperate above pH of 2 and below concentrations of 2 mM.** Wang et al. [61] compared very defective liquid-exfoliated from powder (ce-MoS_2_) nanosheets with averaged lateral dimensions of 250 nm with less defective, ultrasonically exfoliated from powder (ue-MoS_2_) nanosheets. In both cases the edge effects played a crucial role due to small lateral dimensions of the nanosheets of 100–150 nm vs. several microns in typical mechanically exfoliated geological MoS_2_ flakes. Furthermore, the ce-MoS_2_ nanosheets aggregated visibly in water below pH of 1.9 and in concentration of Mo species of 210 ppm Mo (highest out of the three concentrations they prepared), which corresponded to 2.2 mM solution of Mo. In contrast, the ue-MoS_2_ nanosheets remained at least partially dissolved in all solutions (see Figure 4a–e).

Wang et al. found out that in pH of 7 the ue-MoS_2_ nanosheets dissolved only by 5% (of their mass) over 7 days as compared to about 50% mass loss in the case of more defective ce-MoS_2_ nanosheets (see Figure 4g). Thus, single non-defective MoS_2_ flakes should not dissolve in tap/fresh/aerated water, which has a typical pH between 5 and 6 due to dissolution of CO_2_ from air. This outcome has been indeed observed in Reference [42]. However, Zhang et al. [62] obtained needlelike protrusions observed by AFM topography on geological mechanically exfoliated MoS_2_ already after more than 1 h of their incubation in DI water with initial pH of 5.65. The authors have associated them with MoO_3_ hydrates crystallizing from the solution containing molybdate ions. While the appearance of the molybdates is possible a priori, other studies [63,64] have shown that eventual crystallization of insoluble MoO_3_ hydrates occurs at highly acidic conditions, at which, in turn, the MoS_2_ does not want to dissolve. This puzzle needs more studies due to complicated chemistry of molybdates, since the appearance of even monomeric molybdate ions, MoO_4_^2−^, leads to various polymolybdate species depending on the total concentration of Mo and pH. For example, Oyerindea et al. [65] studied solutions of molybdate ions as a function of pH and concentration. At a pH of more than 6, MoO_4_^2−^ ions were dominated at all Mo concentrations. At a pH < 6, several polymeric molybdates appeared. At high Mo concentrations, hepta-, hexa- and octamolybdates became dominant successively as the pH was lowered. Mononuclear species remained dominant below 1 mM. Similarly, Piquemal et al. noted that that monomeric molybdates are predominant at pH > 8 and low concentrations, but oligomeric non-peroxidic species prevail at high concentrations and low pH [66]. Finally, local oxidation of the MoS_2_ crystals has been studied by oxidation scanning probe lithography and suggested their subsequent dissolution in water [67]. In the light of results of Wang et al. [61], water-soluble molybdate ions could have been created therein via electrochemical etching of the MoS_2_ crystals.

Overall, according to the results obtained on defective MoS_2_ nanosheets, their dissolution in water is possible at small concentrations of Mo species and at a pH more than 2. This dissolution decreases substantially with the increasing quality of the MoS_2_ sample, i.e., smaller number of basal plane defects. Following that trend, single non-defective MoS_2_ flakes have been found by some researchers not to dissolve in water [42]. Nevertheless, other researchers reported the opposite effect, i.e., dissolving of mechanically exfoliated MoS_2_ crystals in water [61]. Thus, the subject needs more quantitative research, particularly that various molybdate ions form during the dissolving of the Mo oxide species [68].

In conclusion, regarding oxidation of the MoS_2_ samples at ambient conditions, very slow growth of the passivating Mo oxide layer has been observed on the MoS_2_ powders, likely due to their rough surface with many crystalline edges and defects [31,49]. Furthermore, the appearance of Mo oxides at room temperature, mostly in the form of MoO_3_ (with other oxide forms below a detection threshold), has been confirmed on the CVD-grown defective MoS_2_ monolayers [56]. However, in contrast to the MoS_2_ powders and CVD-grown MoS_2_ samples, no oxidation nor aging of the geological MoS_2_ crystalline flakes has been observed at room temperature. The lack of the passivating Mo oxide layers on single mechanically exfoliated MoS_2_ flakes has been confirmed even in the samples left in air for a year. Single MoS_2_ flakes, however, have been shown to gradually increase their number of sulfur defects when left in air, which, in turn, became oxidized towards substitutional Mo species, but not MoO_3_ [60]. However, some variations in MoS_2_ stoichiometry between even the same kind of mechanically exfoliated samples might also take its toll [69]. Finally, Yamamoto et al. [35] showed—via local micro-Raman studies—that thin MoS_2_ flakes start to show any electronic density changes only above 200 °C. In the light of the aforementioned studies, this might mean that accumulation of defects and oxygenated S vacancies start to become detectable microscopically only above 200 °C.

To complement the aforementioned findings in air, the studies in water at ambient conditions have provided the following conclusions. Defective MoS_2_ nanosheets have been found to partially dissolve in water, particularly above pH = 2 and at concentrations of total Mo of less than 2 mM. Their dissolution products were various kinds of molybdate ions, with monomolybdates prevailing above pH of 6–8, depending on the report. Single non-defective MoS_2_ flakes have been found by some researchers to be stable in water, while other reports showed needlelike protrusions on MoS_2_ crystals left in water for more than 1 h. Thus, more research is needed to resolve this issue.

### 2.2. Microscopic Oxidative Etching between 300 and 400 °C

**Figure 5:**
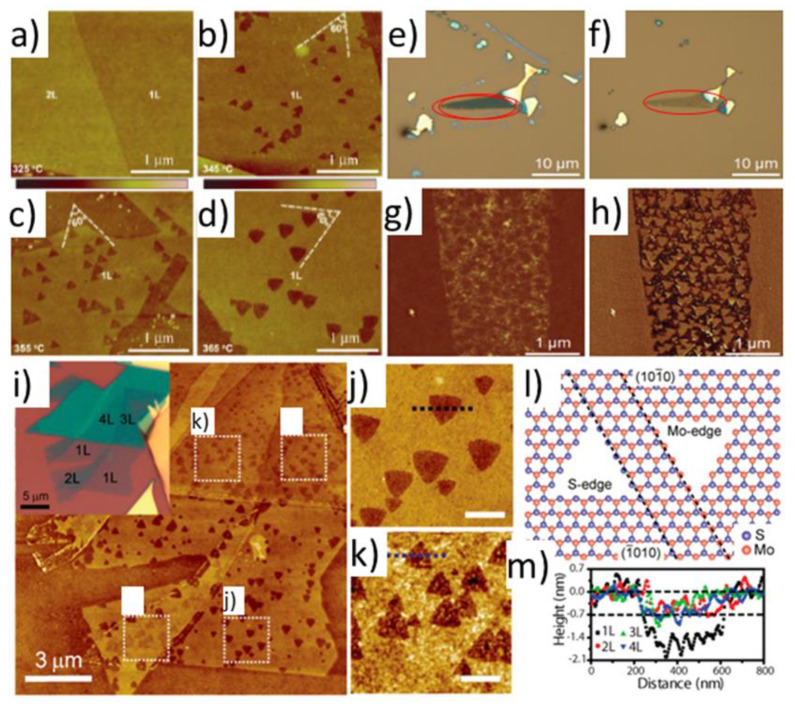
Three seminal studies from 2013 detailing oxidative thermal etching onto basal planes of thin 2H MoS_2_ crystals with emphasis on the formed triangular etch pits. (**a**–**d**) Typical AFM height images showing growth of the equilateral triangular etch pits on a single MoS_2_ flake at sample temperatures of 325, 345, 355 and 365 °C, respectively (each heated for 2 h) [33]. (**e**–**h**) Simultaneous etching and thinning of 2L MoS_2_ nanosheet [34]. (**e**,**f**) Optical image of the flake before (**e**) and after (**f**) thermal annealing at 330 °C for 15 h. AFM height (**g**) and phase (**h**) images of the thinned MoS_2_ nanosheet. (**i**) AFM topography image of a thin MoS_2_ flake annealed at 320 °C for 3 h [35], (**j**,**k**) close-ups on two areas surrounded by dashed lines in the panel (**i**) and differing in the number of MoS_2_ monolayers: 1 L in (**j**) and 4 L in (**k**). They show alternating triangular etch pits between odd and even number of layers. (**l**) Schematic molecular structures of the suggested etch pits. The structure with Mo-edges exposed was suggested to be more stable. (**m**) Height profiles along the dashed lines passing through selected pits confirming their preferential depth of 1 MoS_2_ layer. Parts (**a**–**d**) are reprinted from Reference [33], with permission from Springer Nature Customer Service Centre GmbH: Springer Nature, Nano Research (2013); parts (**e**–**h**) are reprinted from Reference [34], with permission. Copyright ©2013 WILEY-VCH Verlag GmbH & Co. KGaA Weinheim. Parts (**i**–**m**) are reprinted in part from Reference [35], with permission. Copyright 2013 American Chemical Society.

In contrary to no oxidation of the pristine MoS_2_ basal planes below 200 °C, a thermally induced oxidative etching regime has been established in dry air at temperatures between 300 and 400 °C. Such conditions have produced characteristic microscopic triangular etch pits within the MoS_2_ basal planes, as has been observed mostly via AFM. When single MoS_2_ crystals were heated for a short time, the etch pits on basal planes were associated with virtually no change in thickness and lateral dimension of the studied MoS_2_ crystals. Figure 5 summarizes the main results of three seminal papers published in 2013 that were first to report on oxidative etching in thin 2H MoS_2_ flakes heated above 300 °C [33,34,35]. These results were supplemented later by similar one-monolayer deep triangular etch pits on thick 2H MoS_2_ flaked heated above 320 °C [40].

**Figure 6:**
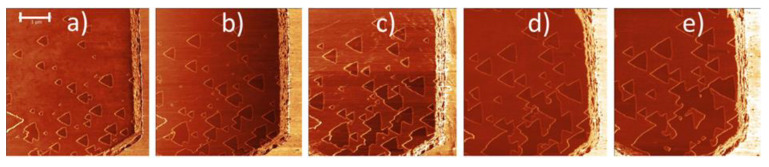
Growth of local triangular etch pits on thick MoS_2_ crystals due to their heating. AFM investigations as a function of heating time on ~30 nm thick single MoS_2_ flake. Scale bar 1 μm. For better visualization, only one-way LFM (∼friction) images are shown. Images from (**a**–**e**) were obtained after heating 10, 15, 20, 25 and 30 min at 370 °C, respectively. Reprinted from Reference [40], with permission. Copyright 2019 American Chemical Society.

Based on the AFM topography images an onset for formation of the triangular etch pits has been observed at 345 ± 10 °C in air [33], 330 ± 30 °C also in air [34] and at 320 ± 20 °C in mixture O_2_/Ar. Temperature errors were estimated by the author of this review not from the precision of the temperature control, which was likely very high in all of these studies, but from the temperature difference between reported data points. A great majority of etch pits were only one MoS_2_ layer deep and originated on single MoS_2_ flakes of 1–9 ML in thickness. Furthermore, they changed orientations in between the subsequent layers (see Figure 5j,k). The same effects at similar temperatures have been observed on thick (more than 10 ML) MoS_2_ flakes (see Figure 6) [40].

**Figure 7:**
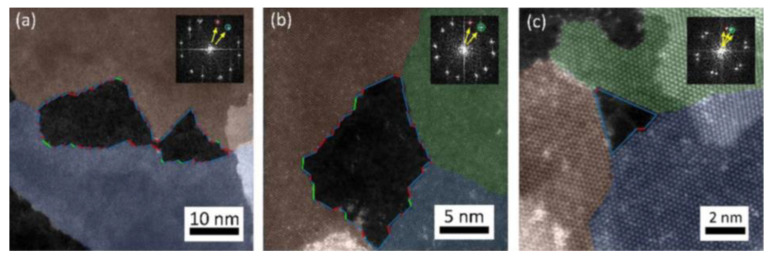
Etching on crystalline edges of the 2H MoS_2_ crystals. (**a**–**c**) ADF-STEM images of etched grain boundaries in as-grown MoS_2_ monolayers. The residual edges are marked with blue, red and green lines, representing zigzag (ZZ) Mo edges, ZZ S edges and ZZ S-Mo edges, respectively. Different grains are labelled by different colors, which are also marked with solid circles in the corresponding FFT patterns presented in insets. Reprinted from Reference [70], Copyright (2017), with permission from Elsevier.

Some variability of the growth speeds for triangular etch pits has been observed between MoS_2_ flakes, which suggested thermally activated processes being at the origin of oxidative etching [35,40]. As it is discussed later, Ukegbu et al. have exploited this observation further to calculate activation energy of the oxidative etching process from the growth rate of the triangular etch pits [40]. The triangular shape of the pits was related to the hexagonal symmetry of the Mo planes within the MoS_2_ crystal lattice, and several mechanisms for their creation have been proposed [33,35,40,70], as is explained later in the review. Furthermore, etching events on crystalline edges were observed by using S-TEM (see Figure 7). Thanks to S-TEM studies, a zigzag Mo (ZZ-Mo) edge was assigned as the dominant termination of the triangular etch pits.

**Figure 8:**
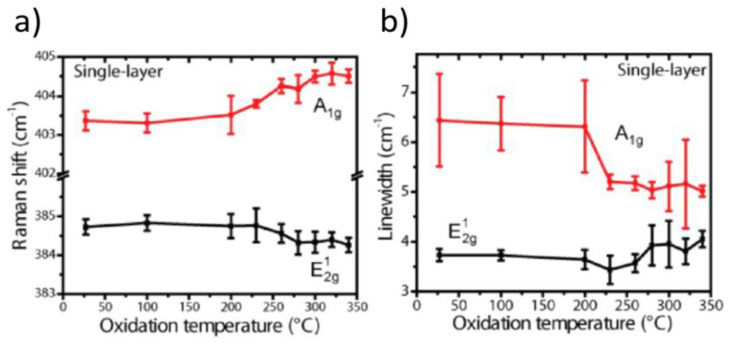
Raman studies of A_1g_ and E^1^_2g_ bands on single-layer MoS_2_ as a function of heating temperature. (**a**) Raman shifts and (**b**) Linewidths of the investigated modes, respectively. Following the trend set by the behavior of the A_1g_ mode, the authors of Reference [35] claimed to observe an electron density withdrawal from the MoS_2_ layer upon oxidative etching. Reprinted from Reference [35], with permission. Copyright 2013 American Chemical Society.

**Raman methods in detecting oxidative etching.** Further details on oxidative etching and triangular etch pits formation have been found using local Raman studies. Current local (usually confocal) Raman setups are easy to use and offer small laterally probed areas with diameters of several microns and less. Furthermore, MoS_2_ flakes display strong Raman bands at 384 cm^−1^ (E^1^_2g_ mode) and 408 cm^−1^ (A_1g_ mode). The mode E^1^_2g_ originates from vibrations of S and Mo atoms within their respective crystal planes, while the mode A_1g_ probes the out-of-plane vibrations of S atoms along the c-axis in a crystal [39]. Chakraborty et al. [71] calculated and measured that any increase of the electron density within single monolayer thick MoS_2_ flakes associated with its oxidation is expected to result in (i) decrease of the linewidth, (ii) increase of intensity and (iii) increase in frequency (blueshift) of the A_1g_ band. They have also reported similar, but smaller changes for the E^1^_2g_ band. Yamamoto et al. [35] observed a decrease of the linewidth and a blueshift of the A_1g_ band upon progressive formation of triangular etch pits within thin MoS_2_ flakes (see Figure 8). At the same time, they observed smaller, but exactly opposite effects for the E^1^_2g_ mode. Their results for the E^1^_2g_ mode were, however, in opposition to the original theory and experiments by Chakraborty et al. Indeed, Yamamoto et al. claimed that the behavior of E^1^_2g_ band upon progressive oxidative etching is unclear. Thus, following the trend set by the behavior of the A_1g_ mode, they attributed observed results to an electron density withdrawal from the MoS_2_ layer and consequently its p-type doping upon progressive oxidative etching.

**Figure 9:**
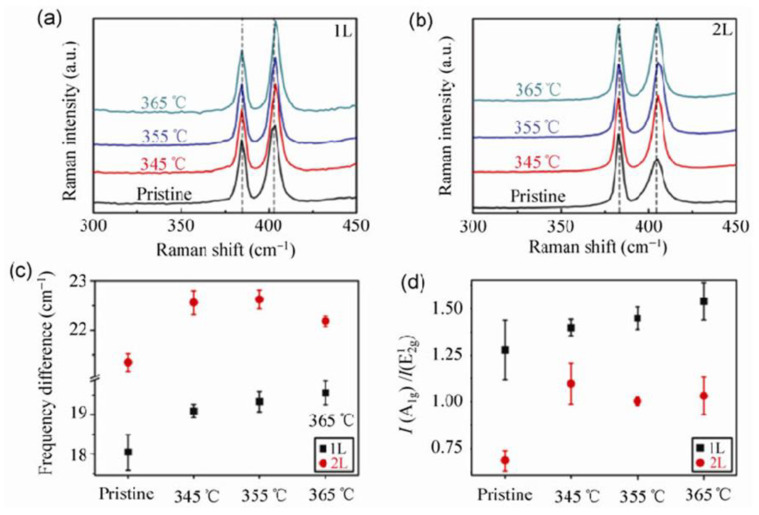
(**a**,**b**) Comparison of the Raman spectra of 1L and 2L MoS_2_ heated in air at different temperatures. (**c**,**d**) The corresponding frequency differences (frequency of the A_1g_ mode minus frequency of the E^1^_2g_ mode) and intensity ratios (A_1g_ mode to E^1^_2g_ mode) for 1L (black) and 2L (red) MoS_2_. Increasing ratio of the intensities in (**d**) corresponds to decreasing ratio of the linewidths. Reprinted by permission from Springer Nature Customer Service Centre GmbH: Springer Nature, Nano Research [33] (2013).

Similar trends of the A_1g_ and E^1^_2g_ Raman bands were observed by Zhou et al. [33] while heating MoS_2_ flakes in air (see Figure 9). They also claimed that the observed effects may originate from the oxygen-induced hole doping, rather than any MoS_2_ lattice deformation induced by thermal etching. The same has been suggested by Wu et al. [34]. However, the hypothesis of the p-type doping of the MoS_2_ layer is complementary to the hypothesis for the formation of small fragments of the p-type MoO_3_ islands on a pristine n-type MoS_2_ surface. In addition, Yamamoto et al., as well as Zhou et al., have shown that frequency shifts of the A_1g_ and E^1^_2g_ modes upon oxidation become negligible for MoS_2_ flakes being thicker than four MoS_2_ monolayers, i.e., 2.5 nm thick. This effect has been explained by the very local nature of the expected changes, where only the basal MoS_2_ monolayer is affected, while at least several MoS_2_ layers are probed by Raman beam. Nevertheless, the authors in Reference [40] performed very careful measurements of the A_1g_ and E^1^_2g_ modes on several MoS_2_ flakes thicker than 10 ML. They observed some differences, but did not collect enough data to explain the observed miniscule differences in the position and width of the collected A_1g_ and E^1^_2g_ modes.

In conclusion, oxidative etching has been so far studied carefully at microscopic scales using AFM methods, S-TEM and Raman spectroscopy. These studies confirmed appearance of the well-formed equilateral triangular etch pits, which grew with temperature and heating time. The also confirmed that such etch pits grew preferential along a zigzag Mo edge termination. Raman studies suggested that within such oxidative regime the MoS_2_ layer became p-doped, which was explained by the oxygen-induced hole doping, but does not exclude formation of the p-doped Mo oxide patches/clusters. Thus, more research is needed to address the origins of the p-doping observed during MoS_2_ oxidative etching.

**Experimentally obtained activation energies for oxidative etching.** There are several relevant experimental studies, which obtained activation energies of the oxidative etching. First, an apparent activation energy, E_a_, for triangular etch pits formation in microscopic MoS_2_ flakes has been experimentally obtained by Rao et al. [37] for thin 1–2 ML MoS_2_ flakes. They obtained E_a_ = 0.54 ± 0.14 eV using the rate of disappearance of the characteristic A_1g_ and E^1^_2g_ Raman peaks associated with the MoS_2_ crystals, which clearly vanish once MoS_2_ crystals disappear. Ukegbu et al. studied thick MoS_2_ flakes [40]. They obtained E_a_ = 1.15 ± 0.25 eV using AFM investigations of the etch pits growth rate obtained at various oxidation temperatures and reaction times. Both of these reports have some shortages. On one hand, some outliers in the data of Rao et al. might be due to non-Arrhenius corrections to the reaction progress from local accumulates of the MoO_3_ species. By accounting on them, an activation energy of 1.09 eV was obtained [40]. On the other hand, Ukebgu et al. estimated the value of E_a_ from an averaged growth of the side lengths of triangular etch pits. However, they did not study etching reactions originating from one and the same node, i.e., one given Mo atom.

**Figure 10:**
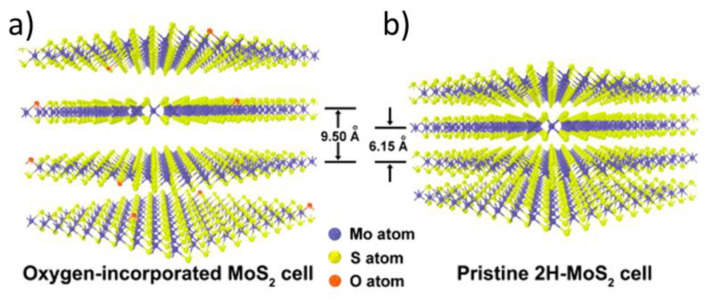
Structural models of (**a**) the oxygen-incorporated MoS_2_ with enlarged interlayer spacing and (**b**) the pristine 2H-MoS_2_. Reprinted from Reference [10], with permission. Copyright 2013 American Chemical Society.

**Additional processes competitive to and/or following up oxidative etching.** In addition to etching two other kinds of interactions with molecular oxygen, which can also follow up oxidative etching, have been observed. In particular, the presence of small MoO_x_ clusters, identified as mostly the MoO_3_ clusters, as well as presence of small MoO_3_ patches [34,42]. These are discussed in detail in the fourth section of this review. Furthermore, other processes have been spotted, such as oxygen diffusion into the freshly exposed etch pits [72] and oxygen incorporation in between the MoS_2_ sheets, which based on the XRD results swells the MoS_2_ crystals (see Figure 10 and References [9,10]). Finally, other kinds of the MoS_2_ etching have been observed via either XeF_2_ [73], He^+^ [74], N_2_ [45] or laser irradiation [75,76].

### 2.3. Microscopic Oxidation above 400 °C

**Figure 11:**
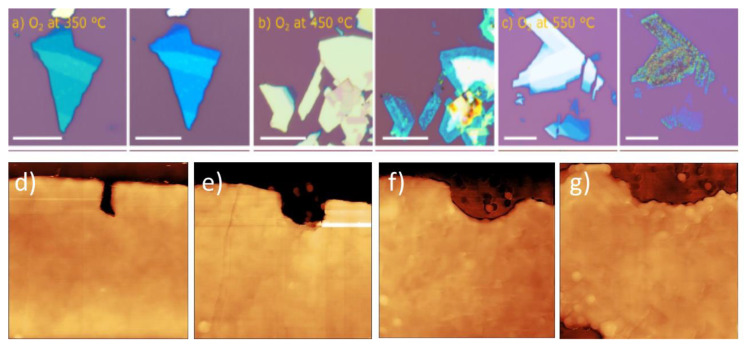
Substantial shrinking of the single MoS_2_ flakes after rapid thermal oxidation in air above 400 °C. (Color online) (**a**–**c**) Optical images of the before (left) and after (right) examples of oxidized flakes previously mechanically exfoliated on Si substrates and annealed in Ar to remove scotch tape contaminants, all flakes heated for 20 min at O_2_. (**a**) At 350 °C, not much etching/oxidation was observed. (**b**) At 450 °C. (**c**) At 550 °C. In each image, the darker regions of each flake are 5–10 layers of MoS_2_ (3.2–6.5 nm), which etch at similar rates. All scale bars are 10 µm. (**d**–**g**) 5 µm × 5 µm AFM LFM images (for better contrast) of a single ca. 20 nm–thick MoS_2_ flake on silica/silicon substrate [77]. The images were collected at room temperature after progressive heating of the sample in air at temperatures and times provided: (**d**) 370 °C at 10 min, (**e**) 400 °C at 5 min, (**f**) 410 °C at 5 min and (**g**) 420 °C at 5 min. Parts (**a**–**c**) are reprinted from Reference [36], with permission. Copyright 2017, American Vacuum Society; parts (**d**–**g**) are adapted from Reference [77].

Several research groups have shown that thin MoS_2_ flakes deposited on silica/silicon either convert (at least partially) into the MoO_3_ crystals or vanish when heated above 400 °C [35,37,42]. In the case of thick MoS_2_ flakes, such oxidation has been associated with substantial crystal volume loss [35,36,40]. Figure 11 presents several examples of such rapid volumetric oxidation.

**Figure 12:**
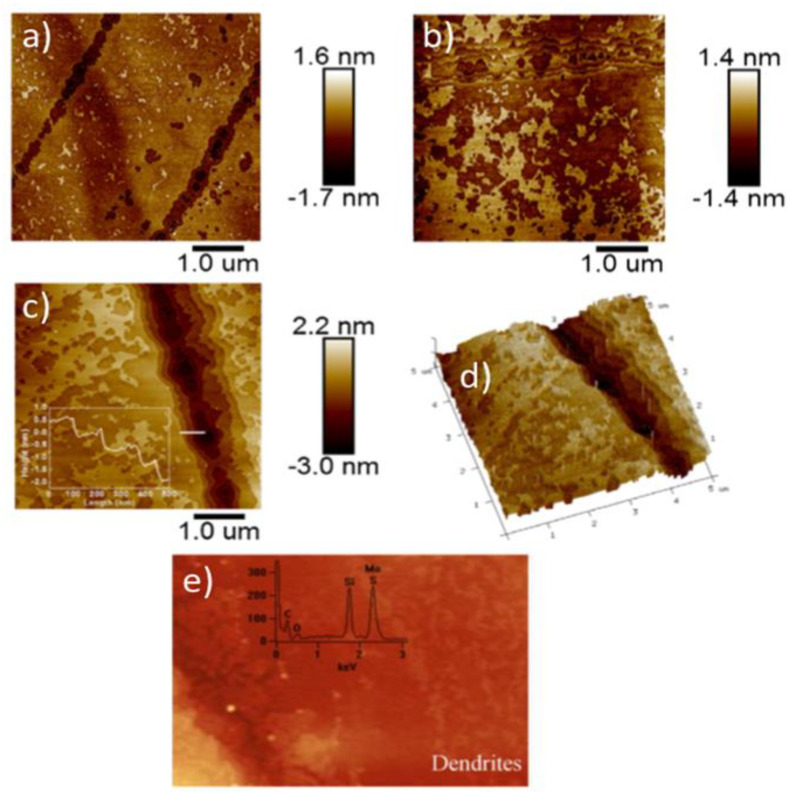
AFM topographical characterization of vigorous oxidation of single MoS_2_ flakes above 500 °C. (Color online) (**a**,**b**) 500 °C in air for 10 min and 1 h, respectively; (**c**,**d**) 500 °C in air for 2 h; (**e**) after several heating rounds of 5–10 min each in temperatures 270–470 °C, followed by a last round at 500 °C. Lateral image dimension of 5 μm. Inset: EDS spectrum after a final round of heating. Visible dendritic structures are present on the MoS_2_ surface. See their discussion in text. Parts (**a**–**d**) are reprinted from Reference [45], Copyright (2017), with permission from Elsevier. Part (**e**) was reprinted from Reference [41], with permission. Copyright 2017 American Chemical Society.

Oxidation at temperatures of more than 500 °C has been shown to be detrimental even to the thick MoS_2_ flakes and led to formation of deep indents and likely Mo oxide patches with messy topographies (see Figure 12) [41,45]. Those were surface morphologies without any clear pit pattern (observed previously in oxidative etching), but rather with many surface pits of various depths and various forms of surface islands with frayed edges. Often, some dendritic structures were observed, as in Figure 12e. The resulting Mo oxides have been shown to leave the substrate surface, but often not entirely if oxidation was not too rapid [35,36,40].

There are clear differences between topographic outcome on the MoS_2_ flakes in the regime of oxidative etching (clean flakes with triangular etch pits; see Figure 5 and Figure 6) and in the regime of vigorous oxidation (messy surface with large topographical variations; see Figure 11 and Figure 12). Thus, one might expect different reaction mechanisms at each of these oxidation regimes. However, based on the recently published literature, we suggest that the messy-looking MoS_2_ surface after its vigorous oxidation can originate from physical blockage of oxidation along particular spatial directions due to locally formed accumulations of the surface-adsorbed Mo oxides and MoO_x_ clusters [42,47,48]. Furthermore, it has been suggested that locally observed dendritic structures, observed on the MoS_2_ samples heated above 500 °C (see Figure 12e) might originate from Mo oxides forming along the directions of the largest concentrations of the initially pre-adsorbed surface oxygen [41]. Nevertheless, in the light of thermodynamic calculations Walter et al. [36] those dendritic structures are rather to originate from rapid surface disappearance of the MoO_3_ oxides above 500 °C due to their conversion of into volatile Mo polymeric oxides, here: Mo_3_O_9_(g) and Mo_4_O_12_(g), which leave rapidly the MoS_2_ crystal surface. Consequently, visible topographic differences between oxidative etching and vigorous oxidations, do not necessarily correspond to a switch between different oxidation mechanisms at these two oxidation regimes [40,41,42]. Those conjectures, however, together with the role of the surface adsorbed oxygen, need stronger experimental proofs as well as relevant theoretical/simulation data.

**“Rapid” oxidation at humid conditions and in water.** One of the first glimpses into the effects of relative humidity on rapid thermal oxidation of single microscopic flakes at local scales have been provided by Walter et al. [36]. They studied bulk single-crystal synthetic MoS_2_ mechanically exfoliated by the Scotch-tape method onto 300 nm SiO_2_/p^+^ Si. Oxygen and oxygen and water vapor (introduced via passing of oxygen through a water bubbler before reaching a furnace) were used to oxidize such MoS_2_ flakes at temperatures from 25 to 550 °C. At a 450 °C heating temperature, Walter et al. calculated very fast etching rates of 40 nm/min for the samples oxidized in oxygen, and up to several times more for analogous samples oxidized in the presence of water vapors. Oxidation at even higher temperature of 550 °C for 20 min resulted in formation of a condensed molybdenum oxide phases containing both MoO_3_ and MoO_2_, as confirmed by Raman spectra. However, those Raman spectra did not show any Raman peaks from the MoS_2_ flakes, thus suggesting that all of the MoS_2_ ions got oxidized within the investigated area, and only the remaining Mo oxide crystals have been spotted. Consequently, no chemical coexistence of Mo oxides and MoS_2_ flakes was demonstrated therein. In another study, Ukegbu and Szoszkiewicz compared percentage of the oxide content onto thick MoS_2_ flakes oxidized progressively from 270 to 500 °C in dry and humid air and using EDS measurements [40]. Moisture has been delivered by placing an open water container present within a heating oven. As expected, the results showed more oxygen and, in consequence, more Mo oxide on MoS_2_ samples oxidized in humid compared to dry conditions. In both cases, however, the likely Mo oxide layers were several nm thick.

According to our knowledge, two other thermal oxidation studies at truly elevated relative humidity have been reported so far. Therein, an enclosed chemical reactor with a controlled pressure of water vapors was used. The first study discussed the coexistence of the Mo oxides onto MoS_2_ flakes oxidized at high relative humidity of 80 ± 7% and at a mean temperature of only 205 °C [42]. The second study discussed a regime of low relative humidity of 9.2 ± 2.2% and heating temperature of 221 ± 8 °C [48]. In both studies, the MoO_3_ layer of thickness of ca. 2 nm has been detected via AFM, KPFM and mechanical scratch tests, as well as Auger spectroscopy. However, a systematic relative humidity-temperature study is needed to validate the hypothesis that oxidation at locally increased relative humidity produces dense MoO_3_ layers onto the MoS_2_ substrates.

It has been suggested that heating above 400 °C and in high relative humidity result in an accelerated production of the MoO_3_ species and their partial conversion to volatile MoO_2_(OH)_2_ both for the MoS_2_ powders and single microscopic MoS_2_ flakes [42]. First, Smolik et al. reported experimental data and thermodynamical calculations on oxidation, volatilization and re-deposition of molybdenum oxide species formed from a certain molybdenum alloy heated between 400 and 800 °C in flowing humid air [53]. The Mo oxide species on the alloy’s surface underwent volatilization, which was detected in the exhaust fumes by the appearance of the MoO_3_ above 550 °C and by the appearance of the MoO_2_(OH)_2_ below 550 °C. Second, Walter et al. calculated that MoO_2_(OH)_2_ is produced in thermal oxidation of the bulk MoS_2_ crystals already at 300 °C (see Figure 3b) [36]. Third, Wang et al. [61] have recently detected that small MoS_2_ nanosheets in water convert partially already at room temperature into the molybdate ions (MoO_4_^2−^), which is also known to be a main product of reaction of MoO_3_ with water and can reconvert into other Mo species [65,66]. It is worth noting that temperatures above 200 °C and high local relative humidity mimic conditions for a hydrothermal MoO_3_ synthesis. Therein, various Mo species have been found to coexist in solution and the outcome was very pH dependent [68]. Consequently, more quantitative research is needed to address the MoS_2_ oxidation at elevated temperatures in humid conditions.

In conclusion, temperatures above 400 °C have been shown to be particularly detrimental to the MoS_2_ flakes. In the case of thin flakes, such oxidation leads to their fast disappearance. In the case of thick flakes, messy surface topographies and various indents, which do not resemble triangular etch pits, observed in oxidative etching have been produced. Water vapors enhance these effects and lead to complete layers of the Mo oxides loosely bound to MoS_2_ flakes. There are still outstanding questions, which have not been resolved, such as origins of dendritic structures appearing on the rapidly oxidized MoS_2_ flakes, and more generally microscopic mechanistic differences between oxidative etching and rapid/vigorous oxidation regimes. Some of them are tackled in the next sections. Finally, understanding of the results for the MoS_2_ oxidation in water and high relative humidity, particularly at a high temperature, is still elusive due to various forms of Mo species known to exist at such conditions, depending on the local pH and large density of defects within such MoS_2_ samples.

## 3. Mechanistic Details of the MoS_2_ Oxidative Processes

In this section, we start with listing the most probable stoichiometry leading to oxidative etching/oxidation of the MoS_2_ flakes in air, humid air and in water. Later, we discuss their mechanistic details based on the published computer calculations, which are mostly local DFT calculations. Apparently only dry oxidation, i.e., without water molecules included, has been computationally studied so far. When available, we provide computationally obtained activation energies (kinetics) and binding energy gains (thermodynamics), as well as compare simulations with existing experimental observations.

### 3.1. Stoichiometric Considerations

Ross et al. suggested that the following overall MoS_2_ oxidation reaction occurs for MoS_2_ powder in humid air:

(1) $$2{\mathrm{MoS}}_{2}+9O_{2}+4H_{2}O\to 2{\mathrm{MoO}}_{3}+4H_{2}{\mathrm{SO}}_{4}$$

Since H_2_SO_4_ is known to be highly hygroscopic, this reaction is expected to be shifted strongly towards the products in water vapors due to local water condensation [29,30]. Indeed, Ross et al. observed more “acidity” than predicted by Equation (1), but attributed it to experimental issues and creation of addition molybdic acid from MoO_3_.

Observations of the 2H MoS_2_ nanosheets in water, together with detection of the arising species, have been performed recently by Wang et al. [61]. They detected that molybdate ion (MoO_4_^2−^) was a main product of the MoS_2_ dissolution in such conditions, according to the following:

(2) $$2{\mathrm{MoS}}_{2}+9O_{2}+6H_{2}O\;\to 2{\mathrm{MoO}}_{4}^{2-}+4{\mathrm{SO}}_{4}^{2-}+12H^{+}$$

Equation (2) points towards partial dissolution of the MoS_2_, which in fact agrees with Equation (1), since molybdic acid was detected originally by Ross et al., and such an acid is the product of the reaction of MoO_3_ with water. However, in light of Equation (1), it becomes questionable whether MoO_3_ is formed first, or rather a direct mechanism of the MoS_2_ conversion into MoO_4_^2−^ in water occurs, Equation (2). It seems that both pathways are possible.

Ross et al. did not provide a formula for an oxidation reaction in the lack of water, but since they discussed only the appearance of the MoO_3_ species, one supposes the following reaction:

(3) $$2{\mathrm{MoS}}_{2}+7O_{2}\;\to 2{\mathrm{MoO}}_{3}+4{\mathrm{SO}}_{2}$$

The SO_2_ is known to convert into SO_3_, particularly at elevated temperatures, which produces a more likely alternative to Equation (3):

(4) $$2{\mathrm{MoS}}_{2}+9O_{2}\;\to 2{\mathrm{MoO}}_{3}+4{\mathrm{SO}}_{3}$$

Consequently, Equations (3) and (4) are the most likely oxidation outcomes in dry air, particularly that MoO_3_ is the most stable of all of the Mo oxides [50]. However, different stoichiometries are also possible. These would lead to the MoO_x_, (MoO_3_)_y_ and SO_x_ species [36]. A good selection of atomistic processes leading to such outcomes is discussed below, with particular stress on their multistep nature. More exotic final products, and in particular, Mo hydrates and Mo hydroxy-oxides, have been suggested to be produced in water [42,62] or when oxidation is catalyzed by water vapors [36,42]. Such Mo hydrates and hydroxy-oxides have not been detected so far on single MoS_2_ flakes, and, according to our knowledge, atomistic simulation approaches leading to these species are currently lacking in the literature.

### 3.2. Computer Simulations

A great majority of mechanistic approaches used atomistic calculations based on the local DFT approaches, which were a primary tool for investigating defect formation energies, reaction energy barriers, reaction mechanisms and the electronic structure properties of the considered MoS_2_ atomistic structures. Typical modeling packages (VASP and/or Quantum-Expresso) differed on the choice of pseudopotentials, periodic supercells, plane-waves, modern exchange and correlation energy functionals, and efficient algorithms to optimize electronic structure, ionic positions and lattice geometries. To compute transition states, the nudged-elastic-band methods were used [78]. They were implemented in the aforementioned packages. Sometimes, ab initio molecular dynamics studies were used to learn about molecular evolution of the pre-obtained intermediate structures. In addition, local functionals and generalized gradient approximations often underestimate reaction barriers due to self-interaction errors [58]. Therefore, obtained reactions barriers should be treated with caution when comparing simulations and experimental details.

**Oxidation studies on the MoS_2_ basal planes.** In the case of the pristine basal MoS_2_ planes, it has been established that initially physisorbed oxygen prefers to sit above sulfur atoms, leading to marginally stable physisorbed oxygen states with an energy gain of only about 0.1 eV, which is comparable with thermal energy of oxygen molecules, *k_B_T*, being 0.05 eV at room temperature [41,43,59,79].

**Figure 13:**
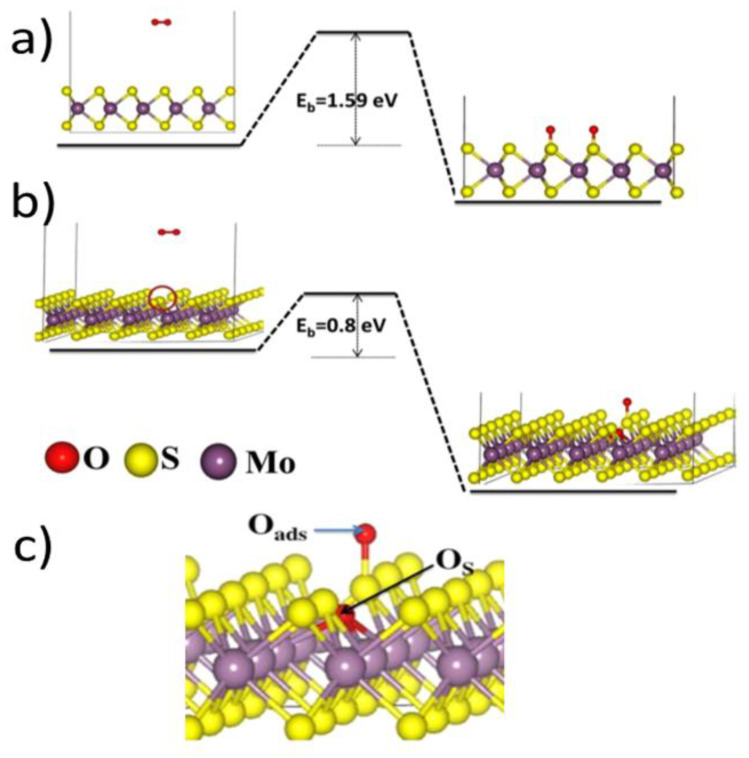
DFT calculations of oxygen dissociative adsorption on MoS_2_ basal plane according to Santosh et al. [43]. (**a**) Pristine (non-defective) MoS_2_ surface resulting in two adsorbed (O_ads_) oxygen atoms on neighboring sulfur atoms; (**b**) S-deficient MoS_2_ surface (SSV) resulting in one adsorbed O_ads_ and one substitutional oxygen (O_s_). (**c**) Close-up on the final state from (**b**) to show atomic positions of O_ads_ and O_s_. Reprinted from Reference [43], with the permission of AIP Publishing.

Regarding engaging into a chemical reaction, the O_2_ molecules, in contrast to molecular oxygen, have been computationally shown to be non-reactive towards the S atoms. A high kinetic activation energy barrier of 1.59 eV was obtained for dissociative adsorption of the O_2_ molecule on 1 ML thick MoS_2_ (see Figure 13a). Dissociative oxygen adsorption leads to adsorbed oxygen atoms in the form of two stable oxygen-terminated sulfurs. This can be considered already oxidation or a step leading to Mo oxides in the meaning of Equations (1)–(4). It is denoted here as follows:

(5) $$O_{2}+{\mathrm{MoS}}_{2}\;\to \mathrm{Mo}{\left(S-O_{\mathrm{ads}}\right)}_{2}$$

Not only is the kinetic barrier for dissociative O_2_ adsorption large, but the process is thermodynamically not efficient, because its binding energy of about 0.8 eV is much less than the kinetic barrier of 1.59 eV [43,57]. Therefore, it is not expected to propagate, and single O_2_ dissociative events are rare.

**Figure 14:**
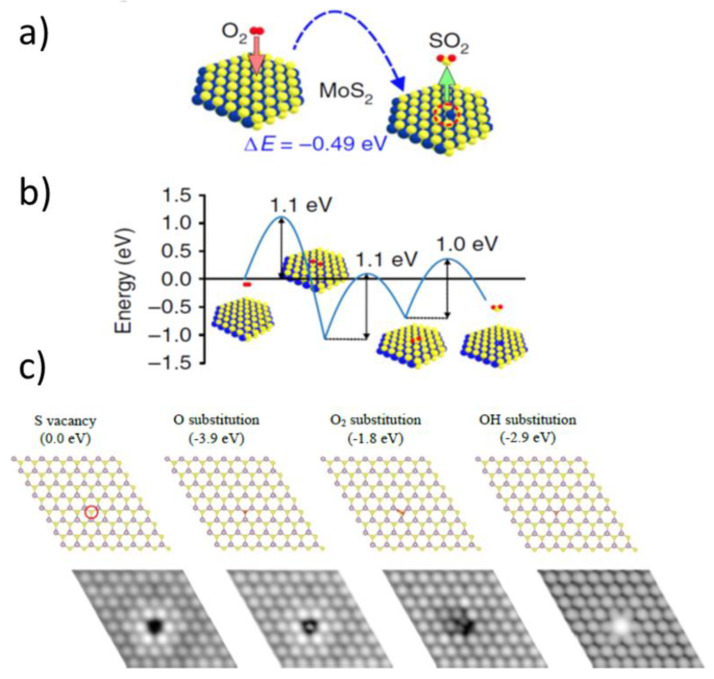
Energetics and kinetics of single sulfur vacancy creation and its later O substitution within the MoS_2_ basal plane obtained by Peto et al. [60]. (**a**) The process of chalcogenide atom vacancy formation through oxidation is characterized by a negative oxidation enthalpy of −0.49 eV. (**b**) Corresponding kinetic energy barriers calculated by the NEB model revealed barriers of ~1 eV. (**c**) O saturation of the SSV yields the ~4 eV energy gain, indicating the highly favorable nature of the O substitution process as a next step, in accordance with experimental STM findings. Reprinted by permission from Springer Nature Customer Service Centre GmbH: Springer Nature, Nature Chemistry [60] (2018).

The findings of Santosh et al. contribute well to explaining the exceptional stability of the pristine, non-defective MoS_2_ flakes against oxidation. Direct oxygen binding to Mo atoms is impossible on pristine basal planes due to steric reasons. However, other oxidative processes have been found to exist on pristine and non-defective MoS_2_ basal planes. For example, oxygen-induced single sulfur vacancy (SSV) creation was observed by Peto et al. [60], who—due to advances in local scale imaging—supplemented their DFT calculations with experimental observations. To start with, they showed an SSV creation by oxygen with enthalpy of such a reaction or formation energy of −0.49 eV (see Figure 14a):

(6) $$O_{2}\;\to \mathrm{SSV}+{\mathrm{SO}}_{2}$$

Despite being thermodynamically favorable, the reaction had to proceed through several stages, but their details were not provided (see Figure 14b). Furthermore, Peto et al. did not find that a created sulfur vacancy would eventually lead to any Mo oxides. However, they suggested that SSV will react easily with atomic and molecular oxygen to form substitutional oxygen species, i.e., oxygen atoms filling the vacancy and becoming directly connected to Mo atoms. The observed process was explained via preserving the original crystal lattice, with the Mo sites in a trigonal prismatic configuration, but coordinated by five S atoms and a substitutional O atom. The mechanistic details for such substitutions were not provided, but the corresponding formation energy for the oxidized vacancy of ~4 eV (see Figure 14c) suggested single oxygen atom substitutions, in accordance with their atomic-resolution STM data. As related, Grønborg et al. studied oxygen exchange on epitaxially grown MoS_2_ single-layer nanosheets [80]. Using a combination of STM and ambient pressure XPS performed in a function of temperature and pressure, they found that such an O exchange was an activated process with an energy barrier of ~0.79 ± 0.20 eV. Atom-resolved STM images reveal O as single defects located on isolated positions on the upper S lattice of MoS_2_, almost in accordance with the results of single sulfur vacancies and their oxidations obtained by Peto et al. [60], who got an apparent activation energy barrier of 1–1.1 eV.

It is noteworthy that the speed for the SSV creation onto the MoS_2_ basal plane under ambient conditions was found by Peto et al. [60] to be of the order of one defect per min per μm^2^. Using the data of Ross et al. obtained for Mo powders in the case of their prolonged oxidation at 100 °C in dry air, one obtains an oxidation speed of about 160 oxidized Mo atoms per minute per μm^2^ [31]. This clearly illustrates different kinds of oxidation mechanisms occurring in these different kinds of the MoS_2_ samples and/or further suggests that macroscopic oxidation of the MoS_2_ powders did not occur on the pristine, non-defected MoS_2_ basal planes.

**Figure 15:**
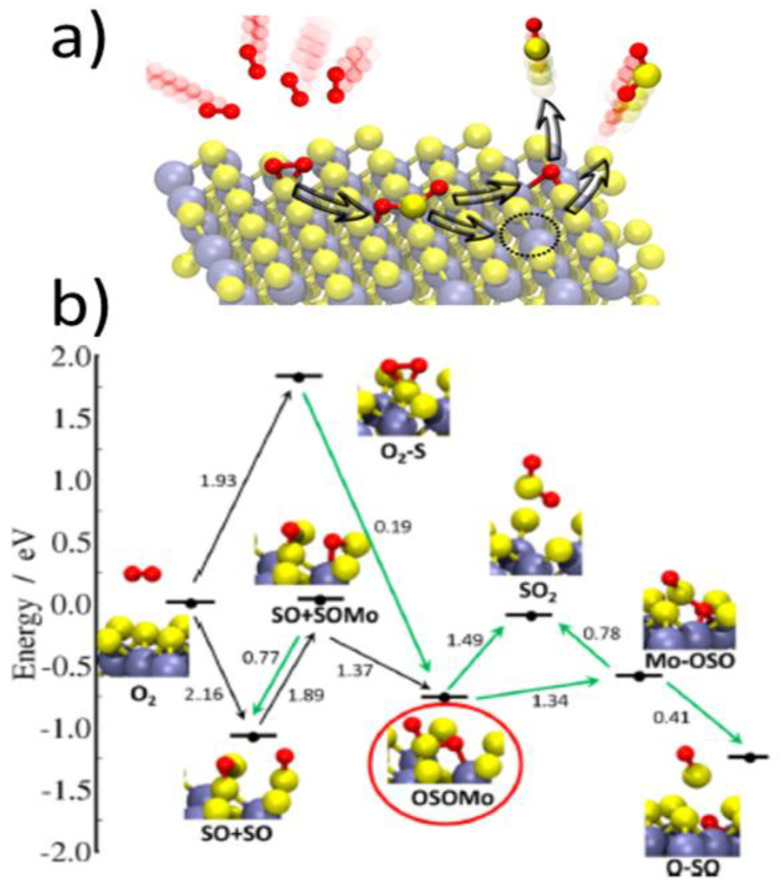
Initial events in oxidative MoS_2_ etching according to Farigliano et al. [72]. (**a**) Initially adsorbed O_2_ molecule (in red) has interacted with the pristine MoS_2_ basal plane to produce the key intermediate consists of an O atom adsorbed on top of an S atom (in yellow) with a second O atom inserted (O_in_) into the S−Mo bond, giving rise to a stable O_ads_−S−O_in_−Mo (OSOMo) moiety. From the OSOMo intermediate, SO_2_ may desorb directly generating a single sulfur vacancy on the surface, while its decomposition leads to the desorption of SO and leaves substitutional oxygen on the surface. (**b**) Activation energies to transition to each intermediate referenced to the reacting O_2_ molecule adsorbed on top of an S atom of MoS_2_ calculated via the cNEB method. The arrows correspond to elementary reaction steps, and the number near each arrow is the activation energy barrier in eV. The green arrows correspond to processes that were observed in the ab initio MD simulations. Reprinted from Reference [72], with permission. Copyright 2020 American Chemical Society.

Recently, a more comprehensive scenario for initial events associated with oxidative etching has been proposed by Farigliano et al. [72] for the non-defective MoS_2_ basal plane (see Figure 15). They used NEB calculations at 0 K temperature to find out crucial intermediates within the processes and then proceeded with ab initio molecular-dynamics simulations at higher temperatures to show decomposition pathways of the key intermediate in the processes. The key initial intermediate consisted of an O atom adsorbed on top of an S atom (O_ads_) with a second O atom inserted (O_in_) into the S−Mo bond, i.e., the O_ads_−S−O_in_−Mo (OSOMo) moiety. Such an intermediate was obtained via two pathways (see Figure 15b). The first pathway proceeded via a dissociative oxygen adsorption pathway on neighboring sulfur atoms with E_a_ of 2.16 eV and its further surface reorganization. Second pathway was a direct O_2_ adsorption on the same sulfur atom with E_a_ of 1.93 eV and its further surface reorganization. However, E_a_ for both pathways are at least 0.4 eV more than obtained via Santosh et al. [43] for dissociative oxygen adsorption onto pristine MoS_2_, which suggests that the trajectory proposed by Farigliano et al. [72] less probable or that there were some major differences in the DFT implementations between these studies. Nevertheless, the results of Farigliano et al. provide an important link between the dissociative oxygen adsorption and oxidative etching. This is because, in the subsequent steps, Farigliano et al. [72] have found out that the OSOMo intermediate decomposes either via direct SO_2_ desorption to generate a single sulfur vacancy, or via SO desorption, leaving substitutional oxygen on the surface.

**Defective basal MoS_2_ planes.** It has been established that single sulfur vacancies in the basal sulfur planes are the most frequent and important from the point of view of an initial oxygen attack [70,81,82]. Therefore, beyond pristine non-defected basal MoS_2_ surface, similar surfaces, but with SSV, have been computationally considered.

The process of dissociative oxygen adsorption occurring on the S-defective sites within an MoS_2_ basal planes has led the calculations of Santosh et al. to a substitutional oxygen, O_s_, bound to Mo atoms, as well as an adsorbed oxygen bound to a sulfur atom [43], O_ads_ (see Figure 13b,c for more details). The corresponding reaction could be denoted:

(7) $$O_{2}+\mathrm{Mo}\left(S\right)\left(\mathrm{SSV}\right)\to \mathrm{Mo}{\left(O\right)}_{S}{\mathrm{SO}}_{\mathrm{ads}}$$

Santosh et al. obtained an energy barrier of 0.8 eV for a process characterized by Equation (7), which is only half of the value obtained on pristine MoS_2_ basal plane. Nevertheless, a much larger value of the obtained binding energy with respect to dissociative adsorption on pristine basal planes, i.e., 1.7 eV energy gain after the reaction, provides a viable thermodynamic argument for this process. In other words, it is still not very probable, but once it occurs, the resulting energy can accelerate the process when other SSVs are in the close neighborhood. Consequently, this could also explain why S vacancies at the grain boundaries in polycrystalline samples accelerate the oxidation process in CVD samples as compared to the monocrystalline non-defected MoS_2_ basal planes. However, dissociative oxygen adsorption does not produce MoO_3_, which has been detected on the mentioned earlier CVD-grown MoS_2_ samples. It can be seen, however, as a first step towards the creation of the MoO_3_ species, as is explained later on in the review.

**Figure 16:**
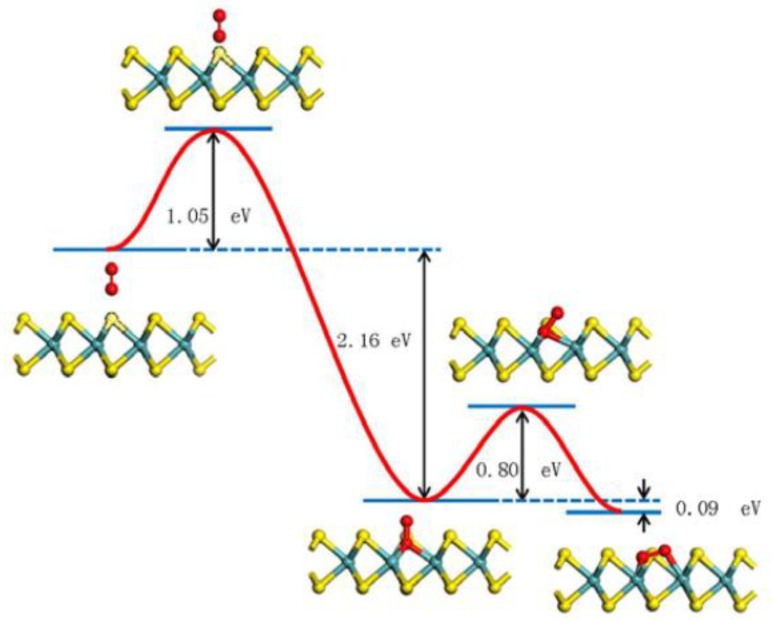
Oxygen dissociative adsorption on MoS_2_ basal with SSV according to Nan et al. [79]. The reaction path consists of two steps. In the first step, the physisorbed O_2_ approaches MoS_2_ sheet and the oxygen atom at the lower end forms chemical bonding with the unsaturated Mo atoms. In the second step, it takes 0.8 eV to accommodate the O_2_ from the standing configuration to the lying-down configuration, which has an energy advantage of 0.09 eV. Reprinted from Reference [79], with permission. Copyright 2014 American Chemical Society.

Another take on the dissociative oxygen adsorption occurring on the S-defective sites within an MoS_2_ basal planes has been published by Nan et al. [79], who, similarly to Santosh et al. [43], used a nudged elastic band (NEB) method implemented within their DFT calculations. They obtained an oxygen molecule substituting two neighboring Mo atoms (see Figure 16). The apparent activation energy barrier of the entire process (1.05 eV) was associated with a binding energy gain of 2.25 eV, which made the process thermodynamically favorable, similar to the results of Santosh et al., who had, however, obtained a much lower energy barrier, and thus provided a more likely reaction pathway.

A direct take on oxidative etching, without involving any dissociative oxygen adsorption, but with an initially defective MoS_2_ crystalline edge has been proposed by Zhou et al. [33]. Their first-principles computations identified the most exothermic reaction path, which proceeded along three independent zigzag directions created by honeycomb assembly of the Mo atoms. Similar conclusions and explanations were provided by experimental works of Lv et al. [70] from high-resolution S-TEM studies (see Figure 7).

**Figure 17:**
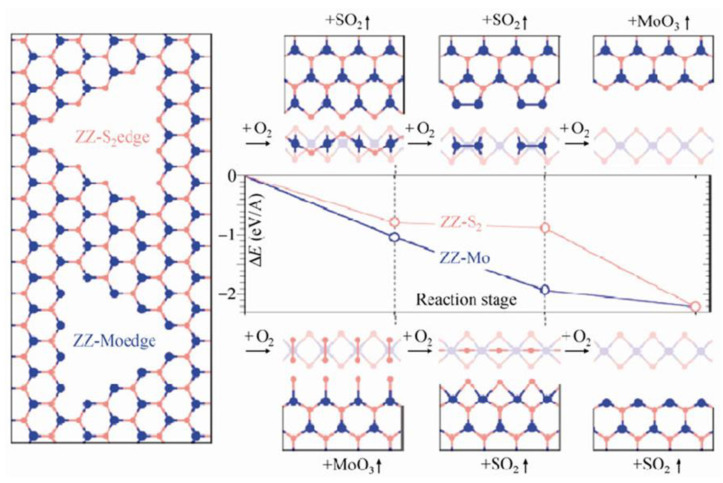
Oxidative etching according to Zhou et al. [33]. An atomic mechanism showing the formation of triangular pits in MoS_2_ layers. A pit in MoS_2_ could generally expose various types of edges, including ZZ-Mo and ZZ-S_2_, as shown on the left. The Mo and S atoms are represented by large (blue) and small (red) circles, respectively. Right: The atomic edge structures (top and side views) during oxidation of ZZ-Mo are shown in the bottom panels, and those for ZZ-S_2_ are displayed in the top panels. ΔE is defined as the enthalpy change (normalized by the edge length) from one edge structure to another, by incorporating O_2_ and evaporating MoO_3_ or SO_2_ gas molecules. Starting from the ZZ-Mo edge (bottom panel), the oxygen reacts first with Mo and then S, while for ZZ-S_2_, the reaction is in the reverse order. Notably, significant structural reconstructions are found in both cases. During the etching of ZZ-Mo, the energy drops monotonically, while for ZZ-S_2_, there is a plateau, suggesting that the ZZ-S_2_ edge probably propagates more slowly than in the ZZ-Mo edge, and thus appears at the etched pit. Reprinted by permission from Springer Nature Customer Service Centre GmbH: Springer Nature, Nano Research [33] (2013).

Zhou et al. presented their mechanistic considerations, along with respective binding energies, E_b_, obtained in each step of the processes they described (see Figure 17). Unfortunately, the values of activation energies for each stage were not provided. Thus, a direct kinetic comparison with the aforementioned models is not possible. The studies of Zhou et al. considered an already formed pit within the basal MoS_2_ plane, which by itself to form is a very costly process with an activation energy per edge atom between 1.59 and 2.19 eV, as provided by Santosh et al. [43] and Farigliano et al. [72], respectively. From such a starting point, the results of Zhou et al. provided a tempting explanation of the oxidative etching. The proposed mechanism fulfilled a generally accepted reaction presented in Equation (3), but involved three stages and significant surface reconstructions after each step. First, an oxygen molecule had to react with unsaturated Mo atoms. A molecule of the MoO_3_ was produced, as well as Mo vacancies with exposed S atoms. Second, the unsaturated and S terminated layer yielded two SO_2_ molecules via two subsequent reaction steps with O_2_ molecules. The reaction was expected to propagate along the C_3_ symmetry within the basal planes of the MoS_2_ crystals to create macroscopically observed triangular etch pits. Since there are two possible starting configurations, i.e., either ZZ-S or ZZ-Mo edges, the authors investigated the two of them and concluded that the reaction progressing along the ZZ-Mo edges is preferential in terms of E_b_ drops along the process.

The results of Zhou et al. can be also applied to defective MoS_2_ basal planes. In addition, other kinds of interactions of oxygen with crystalline edges and grain boundaries in polycrystalline MoS_2_ samples have been studied. Longo et al. [57] have studied oxygen adsorption onto Mo atoms within the MoS_2_ nanoribbons. They considered two different scenarios: armchair and zigzag nanoribbons, with adsorption in the metal sites only, since metal–oxygen bonds are much more thermodynamically favorable than sulfur–oxygen bonds, due to the larger difference in respective electronegativity. They claimed that such oxygen chemisorption was a barrierless process, where Mo dangling bonds were a strong thermodynamic and kinetic driving force. Their results did not refer directly to dissociative oxygen splitting or to oxidative etching.

**Figure 18:**
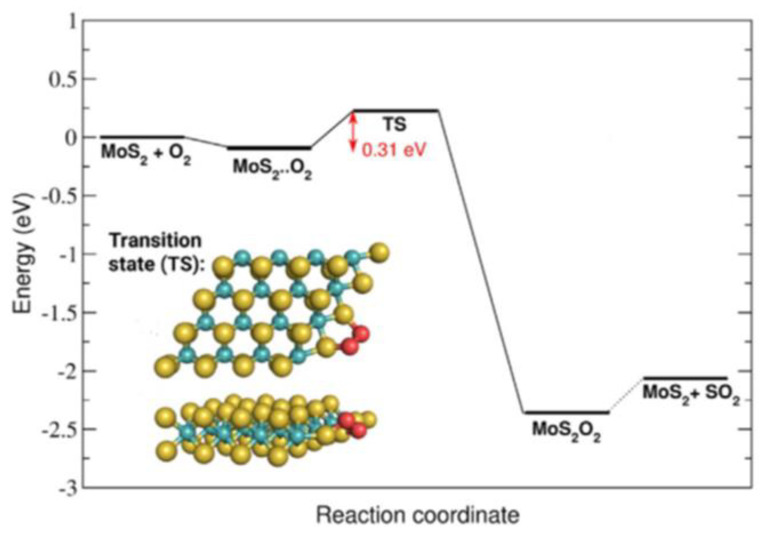
Single sulfur vacancy creation via dissociative O_2_ splitting on the edge of the MoS_2_ crystal [58]. Energy profile for the dissociative splitting of the O_2_ molecule at the Mo-edge of MoS_2_ and the geometry of the transition state (inset). Further reactions leading to single sulfur vacancy are also provided after the transition state. Reprinted from Reference [58], with permission. Copyright 2017 WILEY-VCH Verlag GmbH & Co. KGaA Weinheim.

Martincová et al. have studied oxidative etching directly on “simplified edges” of the MoS_2_ crystals [46,58]. The edge was modelled as a nanostripe cut out from a monolayer of 2H-MoS_2_. It was constructed from S-Mo-S supercells that were only four atoms wide in X and Y directions and long yielding a total of 16 × 3 = 48 atoms. The initial state was taken to be the one in which the O_2_ molecule is in the edge vicinity. The final state of the dissociative splitting was taken to be the one with two separate oxygen atoms adsorbed on adjacent sulfur atoms at an edge. Several dissociation pathways were considered, differing mainly in an initial position of the O_2_ molecule. The most favorable pathway yielded a very low O_2_ dissociation barrier of 0.31 eV (see Figure 18). However, several other pathways yielded similar barriers, all not exceeding 0.5 eV. Such results still do not suggest immediate dissociative O_2_ splitting onto MoS_2_ edges; however, they clearly produce much lower E_a_ values than 0.8 eV obtained by Santosh et al. for the same process on a defective MoS_2_ basal plane.

Very small values of the activation energies obtained by Martincová et al. in the case of edge initiated oxidative etching are expected to produce single reaction events occurring at room temperature over a time scale of μs according to Equation (8) obtained from the transition state theory:

(8) $$t\;\left[s\right]\;\sim \;10^{-12}\;\exp\left(11604\times E_{a}\;\left[\mathrm{eV}\right]\;/\;T\left[K\right]\right)$$

Using Equation (8), one can determine that energy barriers of ca. 1 eV—obtained experimentally in the case of oxidative etching—would correspond to a time scale of a month at room temperature, which is according to what has been obtained for oxidative etching both from simulations and experiments. However, the link between dissociative oxygen adsorption and oxidative etching is not clear. Martincová et al. used a thermodynamic argument that an energy gain of about 2.5 eV in the rate limiting dissociative adsorption was large enough to allow all subsequent reactions to happen. They suggested that after an oxygen molecule dissociated, any adsorbed O atoms (O_ads_) reorganized and started to bind to previously unoxidized sulfur atoms. However, an energy barrier for this process was not provided. Then, in a subsequent step the double substituted sulfur would leave in a form of the SO_2_ molecule with a calculated energy barrier of 0.35 eV for leaving. Exposed Mo atoms would then interact barrierlessly with another oxygen molecule, as in the aforementioned works of Longo et al. [57]. Finally, further oxidation (with energy barriers not provided) would occur through formation of one-dimensional chain-like structures resembling the in-plane (longer) and out-of-plane (shorter) connections between Mo and O atoms in the β–MoO_3_. Such Mo-O structures were bound to respective MoS_2_ edges and facilitated the spread of oxidation onto the surface. Interestingly, the stress associated with the misfit between the MoS_2_ and MoO_3_ lattices made the resulting MoO_3_ structures amorphous [83]. Despite some missing energy barriers, the results of Martincová et al. provide a very good qualitative explanation for the fast oxidative etching rates on edges and defects as compared with basal MoO_3_ planes. They also explain formation of the MoO_3_ and MoO_x_ species on very defective MoS_2_ flakes already at room temperature.

Overall, there are several successes of the presented above theoretical approaches. First, chemical inertness for reactions with oxygen of the non-defective MoS_2_ basal planes at room temperature has been explained in terms of high activation barrier for a dissociative oxygen adsorption onto such planes with an energy barrier of at least 1.6 eV. Second, according to experimental results, oxygen-induced single sulfur vacancy (SSV) creation and its later oxidation have been shown to slowly introduce defects within the MoS_2_ flakes, even at room temperatures, thus making them prone to oxidation via decreasing the energy barrier down to 0.8 eV on SSV and to 0.3 eV on edges/grain boundaries [60]. These results agree qualitatively with experimentally obtained activation energies for oxidative etching which range from 0.6 to ca. 1 eV for pristine MoS_2_ samples, where single oxidation events according to the transition state theory would occur on the time scales of months (see Equation (8)). Third, other oxygen related processes of similar apparent activation energies to oxidation/etching events have been simulated as well. For example, oxygen diffusion into the freshly exposed etch pits [72] or in between the MoS_2_ layers [9,10], which swell the MoS_2_ flakes. However, the experimentally obtained presence of the MoO_3_ species on defective MoS_2_ samples at room temperature was only qualitatively explained [46,58]. Same with faster oxidation/etching on the edges of the MoS_2_ crystals as compared to the basal planes at elevated heating temperatures [35,41,42,46]. Consequently, there are several outstanding questions arising from the theoretical approaches. First, a unified picture for oxidative etching is still elusive. Some models and theoretical simulations refer to dissociative oxygen adsorption on sulfur atoms as an initial limiting step, i.e., studies by Martincová et al. [46], as well as by Farigliano et al. [72], while others refer to direct reaction of oxygen with Mo atoms exposed due to omnipresent single sulfur vacancies, i.e., Lv et al. [70], Santosh et al. [43] and Zhou et al. [33]. In addition, the role of initially physically adsorbed oxygen in thermal etching is still not clear [41], since its diffusion to the reaction centers can compete with direct reactions with atmospheric oxygen. Next, the role of other etching mechanisms such as pure thermal etching without a need for oxygen molecules also needs further theoretical insight [45]. Finally, due to subtle differences within the DFT approaches and varying size and geometries (mostly on edges) of the chosen simulation supercells, there is still room for improvements and new results to explain oxidative etching, as well as the tentative existence of the MoO_x_ species onto various places within the MoS_2_ crystals.

## 4. Mo Oxides and Their Derivatives in MoS_2_ Oxidation

Thermodynamic calculations agree with experimental data that α-MoO_3_ is the most expected MoS_2_ oxidation product in dry and humid air [36,50,51]. However, recent findings confirm that that thicknesses of the MoS_2_ and MoO_3_ single layers are similar [42], which brings up a related question: whether MoO_3_ are present on oxidatively etched MoS_2_ flakes at all, and if yes, then in which form? Figure 2 has summarized current and related phenomenological findings. More details are presented herein.

**Figure 19:**
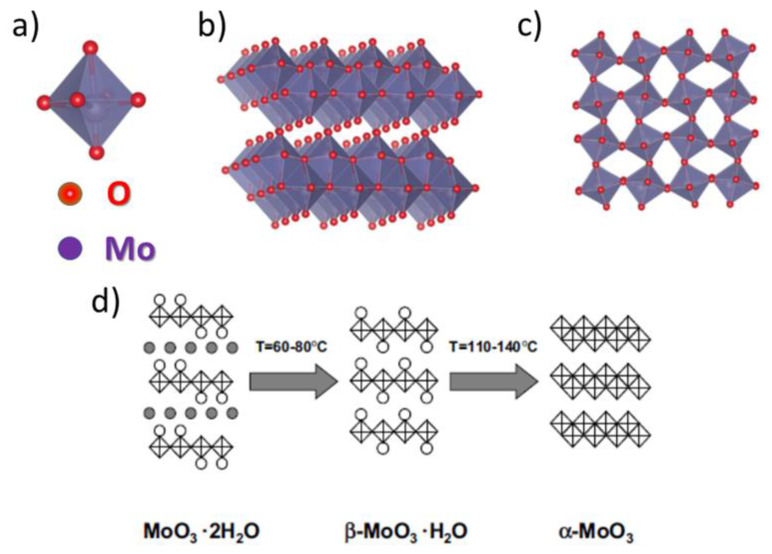
Selected Mo oxide and Mo hydrate species. (**a**) MoO_6_ octahedra as found in the thermodynamically stable α-MoO3 and higher order polymeric molybdate ions. They are composed of molybdenum and oxygen atoms. (**b**) α-MoO3 (molybdite). (**c**) Metastable monoclinic β-MoO3. (**d**) A schematic view of the most popular hydrates crystalline structures of MoO_3_·2H_2_O, β-MoO_3_·H_2_O and α-MoO_3_ [1,2,3,4,5]. Open circles are coordinated water molecules, whereas solid circles show crystal water molecules. Dehydration temperatures are also presented. (**a**–**c**) Adapted from de Castro et al. [50] with permissions. Copyright ©2017 WILEY-VCH Verlag GmbH & Co. KGaA Weinheim. (**d**) Reprinted from Reference [84], with permission. Copyright 2000 IOP Publishing Ltd.

Figure 19 provides some structural information about Mo oxide species. First of all, an orthorhombic α-MoO_3_ is a 2D material, similarly as the 2H-MoS_2_, with layered structure held together by van der Waals forces. Each α-MoO_3_ layer comprises well-connected MoO_6_ octahedra stacked at two height levels. Within each level the MoO_6_ octahedra are connected by shared lateral ends only. Connections between upper and ground levels octahedra are realized by sharing a common edge (see Figure 19b) [51]. Second, Mo hydrates can be seen as derivative species, where water molecules (either in the form of coordinated water molecules or crystal water molecules) have penetrated and destroyed the α-MoO3 structure (see Figure 19d). Finally, in the Mo hydroxyoxides (not shown) coordinated waters from hydrates are substituted by –OH groups.

During oxidative etching (300 to 400 °C), which occurs at isolated spots, the resulting MoO_3_ species are expected to leave into the gas phase. Therefore, their surface presence is not expected. Nevertheless, Gronberg et al. [80] claimed to detect MoO_3_ species on epitaxially created MoS_2_ nanosheets already at about 280 °C. Furthermore, Park et al. demonstrated that CVD-grown MoS_2_ monolayers contain intrinsic MoO_x_ and are quickly oxidized at 100 °C (3 vol% O_2_/He) [47]. In addition, nanomechanical detection of the tentative MoO_x_ species or highly defected MoS_2_ layers was developed on single, non-defective MoS_2_ flakes thermally etched in air at 320–410 °C [42,85]. Finally, very recently, direct chemical proofs for microscale presence of the MoO_3_ nanoparticles and small patched on single MoS_2_ flakes in air during thermal oxidative etching, particularly between 350 and 390 °C, have been provided in separate two reports (one on geological another on CVD-grown MoS_2_) via a combination of local AFM, XPS, TEM, SEM and XAS studies [47,48]. In particular, Park et al. [48] showed that such small MoO_3_ nanoparticles promote oxidation on CVD-grown MoS_2_.

Several reports focused on creation of the MoO_x_ layers under dry and humid conditions as well as in water [41,42,62,67]. However, so far, several contradicting hypotheses for the presence of the Mo oxygenated species in water have been suggested, hinging upon the possibility of spotting various kinds of molybdate ions, molybdic acids, Mo hydrates and Mo hydroxy-oxides [53]. In the case of more vigorous oxidation above 410 °C, some the of the produced MoO_3_ has been found to stay on the surface in the form of patches and layers (see Figure 12), which are very thin and defective due to geometrical mismatch with an underlying MoS_2_ layer [33,34,42]. Finally, the MoO_3_ species have been shown by other, unrelated reports to have a strong tendency to sublimate above 470 °C in air [36] or even at lower temperatures in humid air [42], which should also limit their surface presence. Their thin and likely unstructured counterparts might do so at a lower temperature, and it has been suggested to happen already above 410 °C, in air, for very thin MoO_x_ patches [42].

In order to appreciate the aforementioned studies, one must understand that detection of small amounts of the MoO_3_/MoO_x_ species together with their various morphologies is difficult. The α-MoO_3_ crystals are optically transparent and of similar thickness to single MoS_2_ layers [42,50]. Their presence is easily observed by XPS results [36,42,45,83], but typical XPS studies are not local and report on the MoO_3_ adsorbed on the substrate together with many single MoS_2_ flakes. Furthermore, a vast majority of XPS studies collected on ensembles of single thermally oxidized MoS_2_ flakes at a temperature above 370 °C have exclusively detected the MoO_3_ oxides without any traces of other Mo oxides [42,45,83]. This observation, however, is somewhat expected when Mo oxides are present, since a majority of the XPS studies detect only Mo^6+^ and Mo^4+^ based on Mo 3d electrons. The Mo^6+^ states originate exclusively from the most expected MoO_3_ species. However, the Mo^4+^ states can originate from both MoS_2_ and tentative MoO_2_. Thus, in order to detect MoO_2_ one would need to detect less S^2−^ and more Mo^4+^ as compared to pristine samples. Nevertheless, less sulfur can also be observed when MoO_3_ starts to cover basal MoS_2_ planes limiting electrons originating from sulfur, which reach the XPS detectors. Therefore, MoO_2_ when present at small quantities in relation to large quantities of simultaneously present MoO_3_ will likely be masked in typical XPS studies. Similarly, Micro-Raman studies also lack sensitivity in MoO_3_ detection [40]. According to our knowledge, micro-Raman measurements have not yet delivered any direct proof for the Mo oxide present on single MoS_2_ flakes/crystals as a result of their oxidative etching and oxidations. At best, some authors were able to report Raman shifts only for fully established MoO_3_ crystals [35], while the majority of others could not see any Raman signal from the MoO_3_ (expected at 158, 285, 666, 820 and 995 cm^−1^ in the case of non-resonant Raman excitation) [40,42,48,86]. In addition, both thick MoS_2_ and MoO_3_ are non-magnetic [34]. However, very thin MoS_2_ layers show in-plane magnetism altering in signs between subsequent layers, which produce magnetic contrast between MoO_3_ onto thin MoS_2_, depending on the number of the MoS_2_ layers [34,87,88].

Taking it all together, while MoO_3_ species are locally detected via a combination of several techniques, such as KPFM, nanomechanical studies, XAS, XAS and STEM. However, proper local Mo speciation and surface distribution of other eventual Mo oxides and their derivatives such as hydrates and hydroxy-oxides have not been performed yet.

**The MoS_2_/MoO_x_ interface**. Due to various possible MoO_x_ forms arising on the MoS_2_ surface at various oxidative conditions, the issue of creating and controlling their geometrical dimensions, as well as their chemical composition, becomes intriguing and timely. Pristine 2H MoS_2_ exhibits n-type conduction independent of the metal contact due to a strong Fermi level pinning near the conduction band, promoted especially by sulfur vacancies [89]. However, chemical modification of the n-type MoS_2_ towards p-type MoS_2_ has been reported by using, for example, AuCl_3_ solution [90]. Pristine α-MoO_3_ also exhibits n-type conduction, but with a wide bandgap (>2.7 eV). Nonstoichiometric-reduced MoO_x_ (2 < x < 3) oxides are more conductive, and eventually MoO_2_ is semi-metallic [50]. In addition, thin Mo oxide layers are expected to differ in their electrical properties, depending on their thickness and degree of crystallinity [50,91], and small amounts of α-MoO_3_ can induce p-type doping [92,93]. Therefore, thin Mo oxide patches, if present on the MoS_2_ crystals, are expected to modify their electrical properties and even help in creation electronic p-n junctions in situ, which are nanoscopic in size. A priori eventual boron diffusion from within the typical p-doped boron Si wafers to the Si/SiO_2_/MoS_2_ interface could interfere therein. However, any meaningful boron diffusion to that interface is expected only above 850 °C [94], which is above predicted sublimation of even thick MoO_3_ layers. Finally, a promising new approach to locally create the MoS_2_/MoO_x_ interface would be to use nanolithographic techniques, such as the thermochemical nanolithography (TCNL) [95], local oxidation nanolithography [96] or other lithographic approaches [97,98]. Such methods are expected to produce locally MoO_3_ nanoparticles/thin patches and to chemically reduce any earlier-created MoO_3_ layers. They also have the potential to locally produce active MoO_x_-MoS_2_ junctions of various geometries and chemistries.

## 5. Summary

This review focused on the thermally induced interactions of 2H MoS_2_ crystals with oxygen molecules in ambient conditions. Experimental (Section 2) and computational (Section 3.2) studies investigating oxygen-induced changes to the MoS_2_ structure at various thermal regimes were presented and discussed. Next, the properties and fate of the Mo oxides and other related Mo species arising from thermal oxidation were reviewed (Section 4), along with the properties and tentative ways of creating and modifying the MoS_2_/MoO_x_ interface.

## Data Availability

Data sharing not applicable.
